# Mycobacterium tuberculosis cultured in MGIT media for whole-genome sequencing application: a systematic literature review and meta-analysis

**DOI:** 10.1099/mgen.0.001565

**Published:** 2025-11-24

**Authors:** Emilyn Costa Conceição, Felicia Wells, Abhinav Sharma, Mishka Haffejee, Brendon Mann, Justice Tresor Ngom, Shatha Omar, Johannes Loubser, Miguel de Diego Fuertes, Vincent Rennie, Anzaan Dippenaar, Tim Heupink, Túlio de Oliveira, Gian Van der Spuy, Annelies Van Rie, Robin Mark Warren

**Affiliations:** 1 South African Medical Research Council Centre for Tuberculosis Research, Division of Molecular Biology and Human Genetics, Faculty of Medicine and Health Sciences, Stellenbosch University, Cape Town, South Africa; 2 Tuberculosis Omics Research Consortium, Department of Family Medicine and Population Health, Faculty of Medicine and Health Sciences, University of Antwerp, Antwerp, Belgium; 3 Centre for Epidemic Response and Innovation (CERI), School of Data Science and Computational Thinking, Stellenbosch University, Cape Town, South Africa

**Keywords:** DNA extraction, early positive culture, liquid culture, primary culture, tuberculosis, whole-genome sequencing

## Abstract

Whole-genome sequencing (WGS) holds promise for accurate and comprehensive diagnosis of drug resistance in *Mycobacterium tuberculosis* and identification of transmission events. ‘Early positive cultures’ (EPC) are increasingly used when WGS is implemented to guide clinical care to reduce the turnaround time. We performed a systematic literature review to compare methods used for EPC-based WGS and performed an individual sample data meta-analysis to identify variables associated with bioinformatic quality measures. Of 423 studies identified, 15 met eligibility criteria. We analysed 1,065 FASTQ files from 11 studies using Illumina sequencing; 96.1% passed all quality control thresholds. Median genome coverage was 65× (IQR, 63–82), with a pooled mapping percentage of 91.2%. The meta-analysis showed that the number of sequencing cycles was significantly associated with improved sequencing quality, while other laboratory variables had no consistent effect. Based on these findings, we suggest replacing the term EPC with ‘clinical primary culture’ and propose a standardized workflow and reporting checklist for WGS on primary Mycobacteria Growth Indicator Tube (MGIT) cultures.

**Impact Statement**

Whole-genome sequencing (WGS) of *Mycobacterium tuberculosis* directly from liquid MGIT cultures offers faster detection of drug resistance and transmission than traditional methods, but workflows remain inconsistent across laboratories. We systematically reviewed 15 studies and performed a meta-analysis of over 1,000 sequencing datasets to identify laboratory factors influencing WGS quality. Our results show that sequencing quality depends mainly on the number of sequencing cycles, while other variables have minimal impact. We propose replacing the term ‘early positive culture’ with ‘clinical primary culture’ and present a standardized workflow and reporting checklist for routine use. These findings provide a foundation for reproducible genomic surveillance and accelerate the integration of WGS into tuberculosis diagnostics, particularly in high-burden and resource-limited settings.

## Data Summary

All sequencing datasets analysed in this study were obtained from publicly available repositories and are accessible through the following NCBI BioProjects: PRJEB2858, PRJNA268101, PRJNA302362, PRJNA681718, PRJNA766641, PRJEB21685, PRJNA650381, PRJNA806507 and SRP093599. Accession numbers for all individual samples are provided in Supplementary Table S2. No new sequencing data were generated for this systematic review and meta-analysis. All code, analytical workflows and parameters used for data processing are publicly available, including the MAGMA pipeline (https://github.com/TORCH-Consortium/MAGMA), with additional scripts and methodological details provided in the Supplementary Material.

## Introduction

*Mycobacterium tuberculosis* (*Mtb*) has a slow growth rate and requires up to 60 days (8 weeks) to reach confluent growth on solid media [1,2]. The need for faster methods led to the development of the BACTEC radiometric system (Becton Dickinson Diagnostic Instruments, Sparks, USA) in 1977 [3], followed by the non-radiometric BD BACTEC^™^ MGIT 960 system that is now the global reference standard for *Mtb* culture. The MGIT 960 instrument scans the culture tubes every hour to detect the oxygen consumption of actively metabolizing micro-organisms. The culture is reported as positive when a growth index (GI) of 75 is reached, corresponding to 10^5^–10^6^ c.f.u. ml^−1^.

In the late 1980s, investigators explored the use of early positive liquid cultures for molecular identification methods and showed that this was successful, even when DNA was extracted from MGIT cultures with a GI below 75 [4,7]. Since 2013, there has been a renewed interest in utilizing *Mtb* from liquid culture for drug susceptibility testing of clinical samples using whole-genome sequencing (WGS) [8]. In 2015, Votintseva *et al.* [9] published the first protocol for *Mtb* DNA extraction from ‘early positive cultures’ (EPC).

Despite increasing adoption of EPC workflows, there is no consensus on definitions, optimal incubation periods or standardized laboratory methods for DNA extraction and sequencing. This variability limits inter-study comparisons, slows clinical translation and risks data irreproducibility. To address this, we conducted a systematic review and meta-analysis to assess laboratory variables influencing WGS quality and to propose a new terminology (‘clinical primary culture’, CPC), a standardized workflow and minimal reporting standards.

## Methods

### Systematic literature review

We used Parsifal (https://parsif.al/) for planning and conducting the systematic literature review and adhered to the PRISMA guidelines for reporting [10]. The terms (‘*Mycobacterium tuberculosis*’ OR ‘*M. tuberculosis*’ OR ‘MTB’ OR ‘MTBC’ OR ‘*Mycobacterium tuberculosis* complex’) AND (‘DNA extraction’ OR ‘DNA isolation’ OR ‘Nucleic acid extraction’ OR ‘Nucleic acid isolation’) AND (‘Early positive culture’ OR ‘early positive’ OR ‘early positive liquid Cultures’ OR ‘early positive liquid culture’ OR ‘newly positive liquid cultures’ OR ‘Mycobacterial growth indicator tube’ OR ‘MGIT’ OR ‘liquid culture’) AND (‘Whole-genome sequencing’ OR ‘WGS’ OR ‘Whole genome sequencing’) were used to identify scientific articles published in PubMed (https://pubmed.ncbi.nlm.nih.gov/), BVS (https://bvsalud.org/en/), Science@Direct (http://www.sciencedirect.com) and Scopus (http://www.scopus.com), without restriction on language or publication year. The final search was conducted on 15 January 2024.

Eligibility criteria for inclusion were reported on short- or long-read WGS of DNA extracted from *Mtb* complex isolates. Studies were excluded if they only reported on non-tuberculous mycobacteria (NTM), only included *Mycobacterium leprae*, used methods other than WGS, did not specify the DNA extraction method, did not use liquid MGIT media or performed subculture before DNA extraction.

Two-step eligibility screening of eligible studies was conducted, with an initial review of titles and abstracts by two investigators (ECC and SO), followed by a review of full papers. In instances of disagreement, FW and JTNN performed additional reviews. We also screened the reference section of selected papers to identify possible additional eligible articles. Risk of bias was not formally assessed, but heterogeneity in reporting and missing metadata were acknowledged and considered when interpreting results.

### Data extraction approaches

From the eligible papers, data were extracted on the country where the samples originated from, the study sample size, the MGIT instrument, the GI cut-off, the incubation time after the culture flagged positive, the starting MGIT volume used for DNA extraction, the duration of heat-inactivation time and use of pre-treatment to reduce the human DNA, the DNA extraction method, the DNA quality control (QC) methods used to assess DNA quantity, purity and integrity, the DNA input amount for library preparation, the library preparation kit used, the method used for library preparation QC for concentration (ng µl^−1^) and fragment size (bp) and the platform, analysis and bioinformatics QC used for WGS.

### Standardized assessment of WGS output quality measures

The bioinformatics analysis was performed on the Centre for Epidemic Response and Innovation server at Stellenbosch University. The analysis was limited to the studies using Illumina sequencing, as Oxford Nanopore Technology (ONT) sequencing data are not compatible with the MAGMA pipeline v2.0.0 (https://github.com/TORCH-Consortium/MAGMA).

To standardize the comparison of the WGS results obtained by the different studies, we analysed the publicly available FASTQ files using the MAGMA pipeline [11,12]. Non-*Mtb* reads were not removed prior to analysis by the MAGMA pipeline, as its variant calling workflow is robust to contamination from non-*Mtb* reads.

For each sample, we applied the MAGMA pipeline QC parameters (https://torch-consortium.github.io/MAGMA/customizable-parameters.html). Briefly, the following cut-off values were used: minimum breadth of coverage ≥0.90 (percentage of the reference genome covered by reads), minimum relative abundance of major strain ≥0.70 and a maximum NTM fraction ≤0.20. To maximize the inclusion of samples and ensure a comprehensive comparison across available datasets, the default minimum median depth coverage was reduced from default ≥10× to ≥5× (vertical: number of reads covering a genomic position).

We also calculated the percentage of mapped *Mtb* reads against the total reads, and the *Mtb* genome median coverage adjusted coverage for 5 million reads as a standardized measure to enable robust and comparable assessment of sequencing performance across studies, independent of total reads generated by the sequencing run. Because coverage depth is strongly influenced by the number of raw reads, direct comparison of ‘median coverage’ between sequencing runs or laboratories can be misleading. The adjusted coverage normalizes this bias, allowing fair evaluation of methodological performance in terms of sequencing quality and efficiency.

This metric mainly reflects two biologically and technically relevant parameters, the percentage of reads mapped to the *Mtb* genome and the insert size distribution, which together indicate how effectively a workflow converts reads into usable genomic data. Although mapped percentage can also be reported, it correlates strongly with adjusted coverage, which provides a more comprehensive measure of sequencing yield. Adjusted coverage was calculated as: adjusted coverage=(median coverage/raw read count)×5,000,000. This normalization enables meaningful cross-study comparisons and mitigates artefactual variation from accidental over-sequencing or unequal pooling depths.

For each of these outcome measures, the median and interquartile range (IQR) were estimated for each of the 14 studies that included more than one sample.

### Individual sample meta-analysis

We performed a descriptive analysis of the outcomes of interest (percentage of mapped reads, the median coverage and adjusted coverage for 5 million reads and the percentage of sites represented by a minimum depth of 5 times) using quantile-quantile (Q-Q) plots for each study. We visualized the distribution based on median, IQR and outliers for each of the four outcome variables for each study using boxplot graphs and generated forest plots to summarize the results.

To identify sources of heterogeneity and assess which laboratory methods significantly influenced the WGS quality measures, we applied one-stage random-effects meta-analysis using linear mixed-effects regression models (lme4 package, R v4.4.1) on the available individual sample, with study included as a random intercept to account for clustering. The *P* values were calculated using the Kenward–Roger approximation to estimate the degrees of freedom.

Comparisons included methodologies used for heat inactivation (volume, temperature and time), *Mtb* lysis (enzymatic, mechanical and chemical), purification (bead-cleanup, column-based and chemical-based), library prep (kit’s name and cycles) and sequencing (cartridge, instrument and expected output data).

## Results and discussion

### Studies characteristics

Between 2013 and 2024, 15 eligible studies published results on WGS performed on EPC. Among the 306 unique studies identified, 291 were excluded because they were either a review paper (*n*=39), reported on a study that did not use primary liquid MGIT cultures (*n*=187), did not include *Mtb* cultures (*n*=32) or did not use WGS (*n*=33) (Fig. S1, available in the online Supplementary Material). The 15 included studies were performed in Belgium [13,14], China [15], Ireland, Germany, France and Canada [16], Italy [17], South Africa [18], Switzerland [19], the UK [8,9, 16, 20,22] and the USA [23,25] (Table 1). These results show that the majority of analysed samples are from high-income, low-TB-burden countries, with only two studies conducted in the middle-income, high-TB-burden countries of China [15] and South Africa [18].

**Table 1.** Studies information based on country of origin, sample size, culture incubation time and DNA extraction method

Study reference	Sample country origin	Sample size	Culture incubation time after positivity	DNA extraction method
[8]	Cambridge (UK)	1	Not reported	QIAamp DNA Mini Kit (Qiagen, Hilden, Germany) – modified protocol
[9]	Oxford (UK) (*n*=204), Leeds (UK) (*n*=31)	154	1 to 17 days	QIAamp DNA minikit for DNA purification from blood or body fluids (Spin Protocol) Modified QuickGene DNA tissue kit S for QuickGene-Mini80 [QG] instrument Mechanical cell disruption, DNA Clean up with AMPure XP magnetic beads and ethanol precipitation
[16]	UK, Ireland, Germany, France and Canada	356	Not reported: ‘Newly positive MGIT cultures’	Mechanical cell disruption, DNA Clean up with AMPure XP magnetic beads and ethanol precipitation [9]
[17]	Milan (Italy)	89	Not reported: ‘Early positive routine MGIT tubes’	Maxwell 16 Cell DNA Purification Kit and Maxwell 16 MDx instrument for automated extraction
[20]	Oxford (UK)	27 (23*)	Not reported	Mechanical cell disruption, DNA Clean up with AMPure XP magnetic beads and ethanol precipitation [9]
[23]	New York State (USA)	608	Not reported	InstageGene and Fastprep ZR fungal/bacterial DNA MiniPrep
[21]	Birmingham (UK)	4,156	Not reported, ‘Batched MGIT cultures’	Mechanical cell disruption, DNA Clean up with AMPure XP magnetic beads and ethanol precipitation [9, 16]
[22]	London (UK)	43 (36*)	Not reported	Mechanical ribolysis (FastPrep24 platform) followed by Diasorin IXT (Arrow) automated platform
[18]	London (UK) and Durban (South Africa)	UK (*n*=12) and South Africa (*n*=21)	Not reported	Mechanical cell disruption, purified with DiaSorin Liaison Ixt (DiaSorin, Italy) or cetyltrimethylammonium bromide (CTAB)
[19]	Lausanne (Switzerland)	21 (3 samples from 1 patient) (7*)	Not reported	QIAmp mini Kit (Qiagen, Hilden, Germany)
[25]	New York (USA)	431	Not reported	InstageGene and Fastprep [23]
[13]	Belgium	306	Not reported	MagCore^®^ Genomic DNA Bacterial kit and MagCore^®^ auto-extraction instrument
[13]	Belgium	241	Not reported	MagCore^®^ Genomic DNA Bacterial kit and MagCore^®^ auto-extraction instrument
[24]	New York (USA)	1,779	Not reported	InstageGene and Fastprep modified from Shea *et al.* [23]
[15]	Shanghai (China)	211 (182*)	Not reported	CTAB

*, Analysed in this study.

### Terminology evaluation and proposal

All 15 included studies performed sequencing directly on the primary MGIT culture, i.e. without a subculture step, but none performed DNA extraction immediately after MGITs flagged positive. Instead, most studies incubated samples (for an undefined time) until a large enough batch was available for sequencing. Only one study explicitly defined EPC as 1–7 days after positivity [9].

Being that EPC is vaguely defined as DNA extraction soon after MGIT positivity. The term EPC is inconsistently applied, which limits comparisons; we therefore suggest replacing the term EPC with CPC. CPC definition includes any workflow using the first MGIT-positive culture, with a short re-incubation (0–7 days) before DNA extraction, which captures real-world practice. This distinction is important, as a subculture results in a substantially longer turnaround time and can lead to loss of diversity and failure to detect heteroresistance [26]. Future research should investigate the optimal balance between prolonging MGIT growth for increased DNA yield and minimizing turnaround time.

### Duration of MGIT incubation and wash before DNA extraction

In Table 1, we present the details of all methodological approaches taken and reported by each of the 15 studies included. One study reported that *Mtb* cultures were heat-inactivated between 0 and 17 days after culture positivity (median, 3 days; IQR, 2–5 days), with all remaining cultures negative at 49 days [9]. The other 14 studies did not provide information on GI cut-off used or the days of incubation after culture positivity before heat inactivation for DNA extraction (more details within Table S1).

Some studies [9,16, 18, 20,22] demonstrated that treating MGIT culture samples with saline washing before DNA extraction was implemented to remove contaminating soluble DNA (residual human DNA or DNA from commensal organisms). Such treatment was recommended because it is known that the sputum decontamination process with *N*-acetyl-l-cysteine–sodium hydroxide (NALC/NaOH) liquifies and lyses most human and bacterial cells, leading to co-precipitation of the lysed cells and their DNA with *Mtb* bacilli during subsequent centrifugation. The pelleting of the so-called contaminant does not interfere with *Mtb* growth but remains in the MGIT tube and, if not removed, will be sequenced along with the purified *Mtb* DNA, thereby impacting the quality and read depth of the *Mtb* [9]. In this review, only 46.67% (7/15) of the studies reported including a pre-treatment step before DNA extraction, which primarily followed the protocol proposed by Votintseva *et al.* [9].

### DNA extraction and QC

The volume of MGIT culture used varied from 1 to 8.4 ml (the entire MGIT tube). None of the 14 studies that only used part of the MGIT tube content specified whether the bacilli were allowed to settle or whether the aliquot was taken after vortexing to homogenize the culture. Most studies (*n*=9, 60%) heat-inactivated cultures at 95 °C for 15 min to 2 h. Seven (47%) studies removed human DNA before DNA extraction using a saline wash or the MolYsis Basic5 kit (Molzym, Bremen, Germany); the remaining studies did not state whether they performed a human DNA removal step.

A variety of approaches were used for DNA extraction, including the in-house method published by Votintseva *et al.* in 2015, the manual CTAB method and a range of commercial kits with or without mechanical disruption and with or without the use of an automation instrument. None of the studies reported the volume of total DNA resuspended or the results of an assessment of DNA purity and integrity. To measure DNA quantity, the Qubit dsDNA kit, Qubit fluorometer v2.0 (Thermo Fisher Scientific, Waltham, USA) was most commonly used; a real-time quantitative PCR (qPCR), the QuantiFluor dye system (QuantiFluor dsDNA) (Promega, Madison, USA) and the Quantification Quantus^™^ Fluorometer (Promega, Madison, USA) were less frequently applied.

The DNA extraction process is critical for successful WGS and consists of several essential steps: disruption of the *Mtb* cell wall, release of the nucleic acid and purifying mycobacterial DNA. Unlike other micro-organisms, mycobacteria have a rigid cell wall abundant in lipids, thereby making the bacilli resistant to certain lysis buffers. Selected methods for cell wall disruption include mechanical (using bead-beat in a high-speed homogenizer such as FastPrep), chemical and enzymatic (CTAB) [27,28].

This review found that numerous different DNA isolation methods were used, involving mechanical cell disruption, either alone or combined with physical, chemical or enzymatic approaches. Notably, the ‘gold standard’ CTAB protocol [29] was only reported in two studies. While this method is commonly favoured in the literature for its ability to produce high-quality DNA with large molecular weights, it does have the drawback of being labour-intensive, making it challenging to implement in routine high-throughput laboratory settings. As an alternative, researchers have explored different approaches using commercial kits (such as QIAmp mini kit, IG/FP, MagCore^®^ Genomic DNA Bacterial kit, Maxwell 16 Cell DNA Purification, modified QuickG and Quick-DNA Fungal/Bacterial Miniprep Kit) to reduce the turnaround time for WGS applications.

In the context of next-generation sequencing (NGS) applications, extracted DNA is commonly put through a rigorous assessment of purity, integrity and quantity [27,30]. These factors substantially impact the reliability and quality of the data, specifically sequencing depth and cluster density. Genomic dsDNA is typically quantified in ng µl^−1^ using the Qubit fluorometric instrument (ThermoFisher Scientific, Waltham, USA). Purity is assessed using a spectrophotometer, while DNA integrity is evaluated using agarose gel electrophoresis or specialized microfluidic devices like the TapeStation (Agilent Technologies, Santa Clara, USA).

Despite the importance of proving DNA QC, we noticed that none of the reported studies included data on DNA purity or integrity; instead, they solely focused on DNA quantification. The omission of reporting these metrics could be attributed to either the low volume of resuspended DNA available for analysis or the authors may have considered DNA quantification to be the only essential QC step needed before commencing genomic library preparation. This aligns with the importance of determining the DNA concentration and normalizing the DNA input before library preparation.

### Library preparation

For genomic library preparation, most studies (*n*=12, 80%) utilized the Nextera DNA XT Sample Kit (Illumina Inc., San Diego, USA) or the NEB Next Ultra II DNA kit (New England Biolabs, Ipswich, USA), which are specifically designed for low (≤1 ng) DNA input. One study used the TruSeq DNA Nano Kit (Illumina Inc., San Diego, USA), which requires 200 ng DNA input. Three studies reported normalization for the DNA input to 1 ng (0.2 ng µl^−1^ in a volume of 5 µl). The concentrations of genomic DNA, when reported, ranged from 0.04 to 0.1 ng µl^−1^. When reported, the number of PCR cycles was always 15. Fragment sizes were only reported by two studies: a median of 627 bp (IQR, 495–681 bp) or a mean of 717 bp (range, 650–1,200 bp).

Ideally, the library preparation protocol and QC steps should be easy to perform, rapid, reproducible, economical and achieve good quality libraries for sequencing of *Mtb* on CPCs. The Nextera XT library preparation kit was the most frequently used library kit, probably due to the recommended DNA input of 1 ng. This kit also enabled libraries to be successfully generated with an input DNA amount as low as 0.043–0.3 ng µl^−1^ [9,19]. Using these amounts, the mapped reads against the reference were of sufficient depth coverage for reliable variant calling in all isolates [19]. The NEBNExt Ultra II DNA kit was used by certain workflows based on the low DNA input requirements when compared to the TruSeq DNA Nano Library Prep Kit, which requires 200 ng of input DNA [31,33]. The popularity of the Nextera XT library preparation kit [34] also relates to the shorter workflow compared to the NEBNExt Ultra II DNA kit and the TruSeq DNA Nano library preparation kit. Another reason for the Nextera XT’s popularity is the lower cost for the kit itself (excluding sequencing reagents, labour, culturing and gDNA extraction costs), compared to the NEBNExt Ultra II DNA kit, which needs additional reagents [32], and the TruSeq DNA Nano library preparation kit, which requires specialized equipment, such as a mechanical fragmentation device (Covaris) [33].

Library preparation efficiency can be enhanced by increasing either the input DNA or PCR cycles. While increasing input DNA is typically beneficial, it’s often impractical for CPC samples due to limited gDNA yield when extracted from CPC cultures. Our analysis indicates that PCR cycles of the library preparation step were the primary factor affecting mapped percentage and adjusted coverage per 5 million reads (coefficients±sd=0.136±0.058 and 0.542±0.120, respectively). Accordingly, optimizing PCR cycles was the most effective strategy to increase the library yield, especially when working with low DNA input (<1 ng).

Only two studies reported their library QC data [19,20], highlighting a significant knowledge gap in the literature for CPC sequencing. Importantly, the number of libraries pooled directly influences the coverage depth of each sample, and it is essential to report on the number of pooled libraries per flow cell type [35]. Overall, eight studies reported on the number of pooled samples [9,16, 18, 20, 21, 23,25]; however, one study only reported the theoretical coverage depth [13]. Moreover, the type of sequencing kit also contributes to the sample coverage depth, which is linked to the sequencing data output. The number of samples pooled defines the coverage depth and thus is directly linked to the amount of funding available.

### Sequencing methods

All studies used Illumina (short read) sequencing, and two studies also used ONT (long read) sequencing. The number of libraries per flow cell varied from 9 to 17 when using the MiSeq V2 300 cycle kit (2×150 bp paired-end) and 12 to 18 (including controls) when using a MiSeq V2 500 cycle kit (2×250 bp paired-end). The number of samples processed was 18 for the MiSeq V2 500 cycle kit (2×250 bp paired-end) or 48 for the NextSeq 500 mid-output 300 cycle kit [18,23]. Four papers did not list the sequencing kit chemistry used. The Illumina MiSeq platform was most frequently used (*n*=13); other platforms included Illumina MiniSeq, NextSeq 500/550, HiSeq and ONT MinION.

ONT has potentially a simpler laboratory workflow than other sequencing technologies, making it favourable for decentralized sequencing. However, it requires a high amount of input DNA, at least ≥50 ng. Currently, ONT library preparation kits include Ligation Sequencing, Rapid Sequencing and Rapid PCR barcoding kits, each with a recommended gDNA input range of 1,000, 50–100 and 1–5 ng, respectively. Among the studies we reviewed, Votintseva *et al.* [20] used ONT, but not specifically for CPC samples. Instead, they applied it to technically produced spiked sputum samples (BCG-spiked in Ziehl Nielsen negative sputum) with low DNA concentration. The MinION sequencing was performed using R9/R9.4 flow cells and PCR-based sample preparation. They utilized 20 ng of DNA as input for the PCR, resulting in a final amount of 43 fmol loaded onto the MinION flow cell. Subsequently, Smith *et al.* [25] adopted a similar ONT protocol for CPC samples, using a maximum of 20 ng DNA as the template and the ligation sequencing kit (catalogue no. SQK-LSK109). In this case, ~130 fmol of the final libraries were loaded onto the MinION flow cell version R9.4.1. These studies demonstrate the promising potential of using ONT in CPC samples. However, a systematic and technical study should validate its performance in samples with low DNA concentrations, comparing it with those from solid cultures or subcultures with high DNA concentrations.

### Meta-analysis of WGS quality parameters

A meta-analysis of 1,065 FASTQ files from 11 studies using Illumina sequencing technology (Table S2) revealed that 1,023 files (96.1%) met all QC thresholds. Pooled estimates across studies showed a median genome coverage of 65× and a mapped read percentage of 91%. Failures were largely attributable to insufficient coverage (<5×) or breadth (<0.90). This indicates that the majority of the studies generated high-quality data with satisfactory coverage and mapping rates, supporting the suitability of these samples for routine WGS-based diagnostics, even in the presence of certain levels of non-*Mtb* DNA contamination. However, some studies showed a high proportion of samples fail QC, highlighting the importance of consistent lab procedures.

The QC failures were due to criteria such as coverage, breadth and relative abundance issues (*n*=16), inadequate coverage and breadth (*n*=14), coverage and relative abundance (*n*=6), low coverage (*n*=4) and insufficient NTM fraction (*n*=2). The analysis also showed that the number of sequencing cycles was positively associated with data quality (mapped percentage *P*=0.008 and adjusted coverage per 5 million reads *P*<0.001), while other factors like heat inactivation and DNA purification methods did not significantly impact quality. The studies with samples failing QC included Votintseva *et al.* 2017 (*n*=19 of 59, 32.20%), Doyle *et al*. (*n*=15 of 71, 21.12%), Votintseva *et al.* 2015 (*n*=5 of 54, 9.25%), Shea *et al.* 2017 and 2021 (*n*=1 of 396, 0.25%), Pankrush *et al*. (*n*=1 of 21, 4.76%) and Wu *et al*. (*n*=1 of 182, 0.54%). The datasets from Shea *et al.* [23, 24] were analysed together within a single BioProject, as there is no distinction or separation between the datasets based on the year of publication. Conversely, 100% of their samples from Bogaerts *et al.* (*n*=241), Koser *et al.* (*n*=1), Zakham *et al.* (*n*=7) and Nimmo *et al.* (*n*=33) had passed QC.

**Fig. 1.**
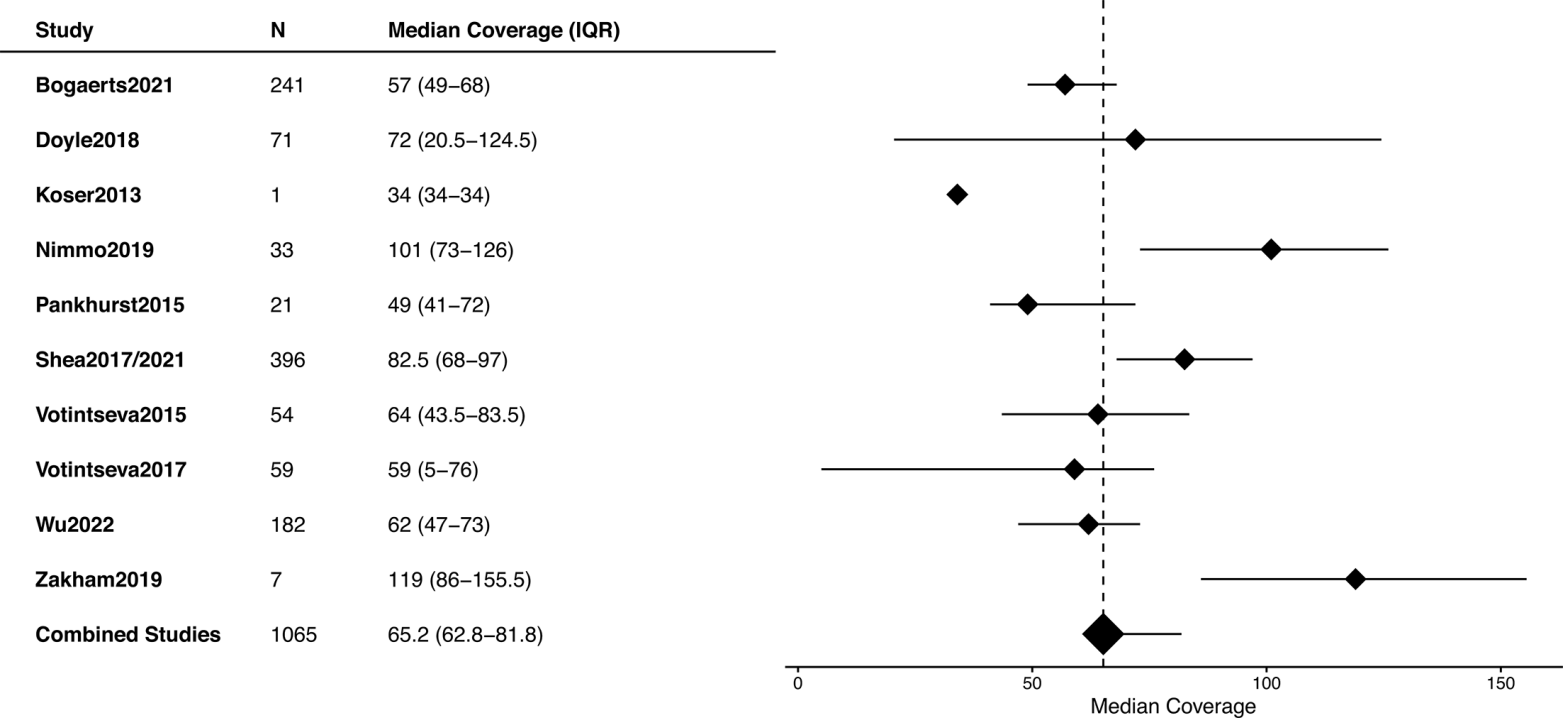
Distribution of median genome coverage (vertical depth: number of reads covering a genomic position – site), for each study included in the meta-analysis.

**Fig. 2.**
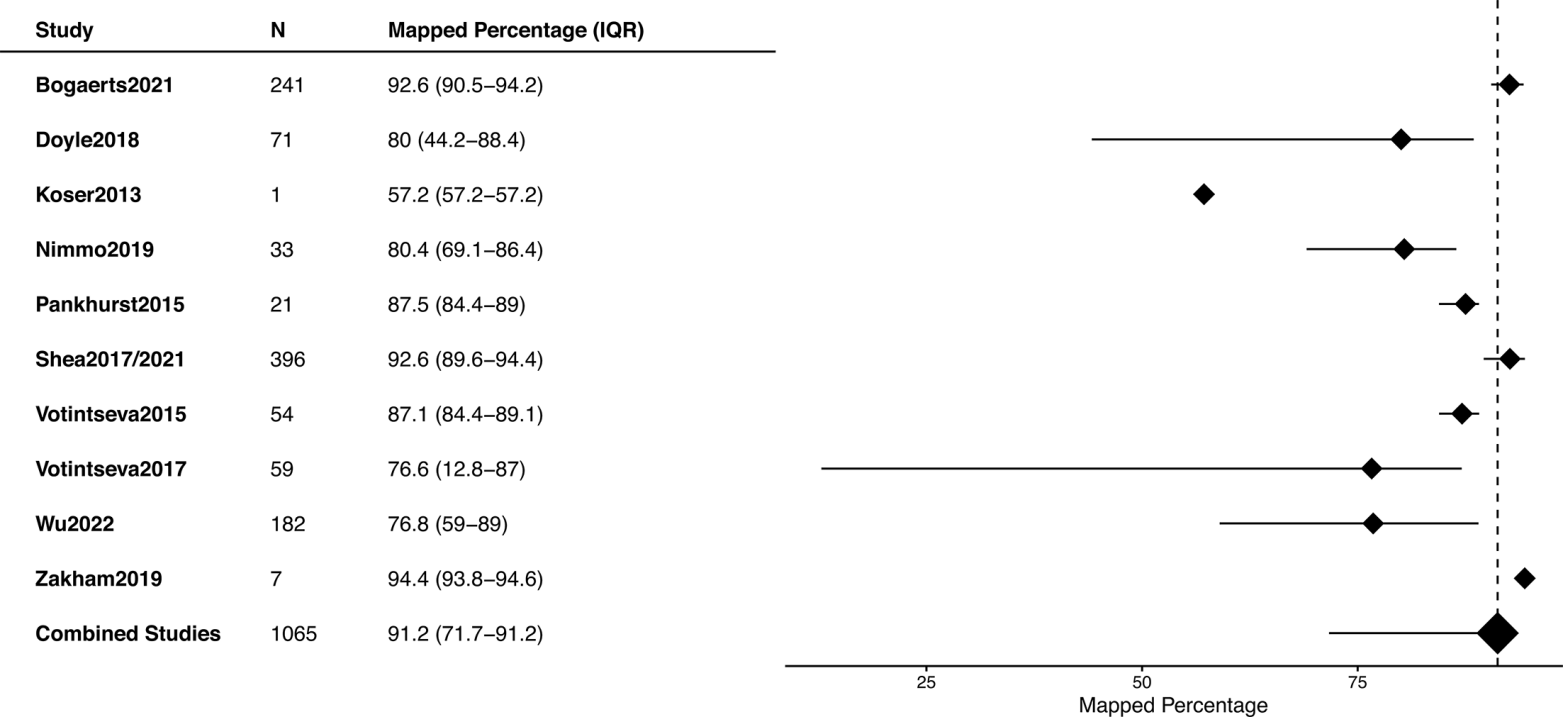
Distribution of percentage of mapped *Mtb* reads against the total reads, for each study included in the meta-analysis, considering the reference H37Rv.

Across the studies, the median genome coverage ranged from 50× to 100×, with a pooled estimate of 65.2 (IQR, 62.8–81.8) (Figs 1, S2 and S3). The percentage of mapped reads was consistent across studies, varying between 75 and 80%, with a pooled estimate of 91.2% (IQR, 71.7–91.2) (Figs 2, S4 and S5).

**Fig. 3.**
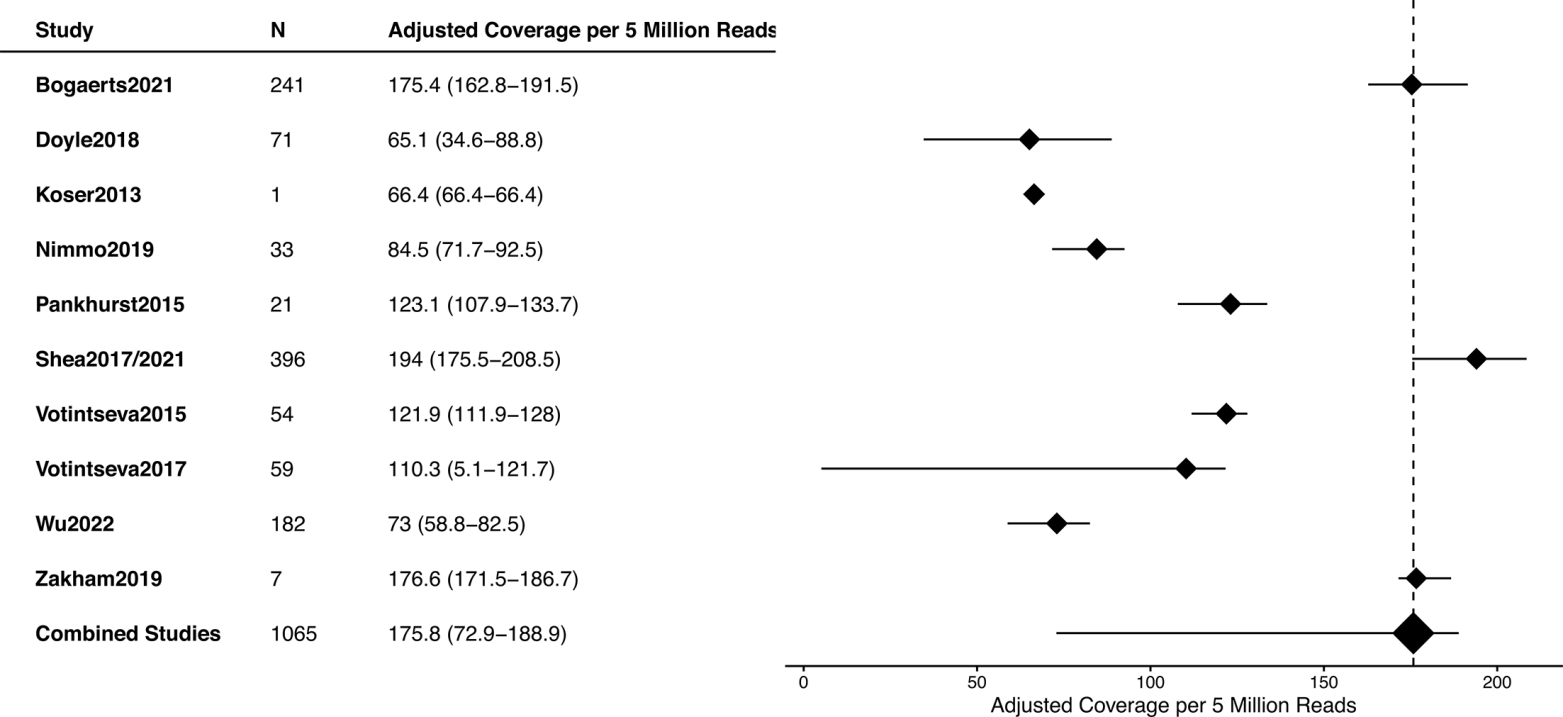
Distribution of adjusted median depth coverage per 5 million reads, for each study included in the meta-analysis.

**Fig. 4.**
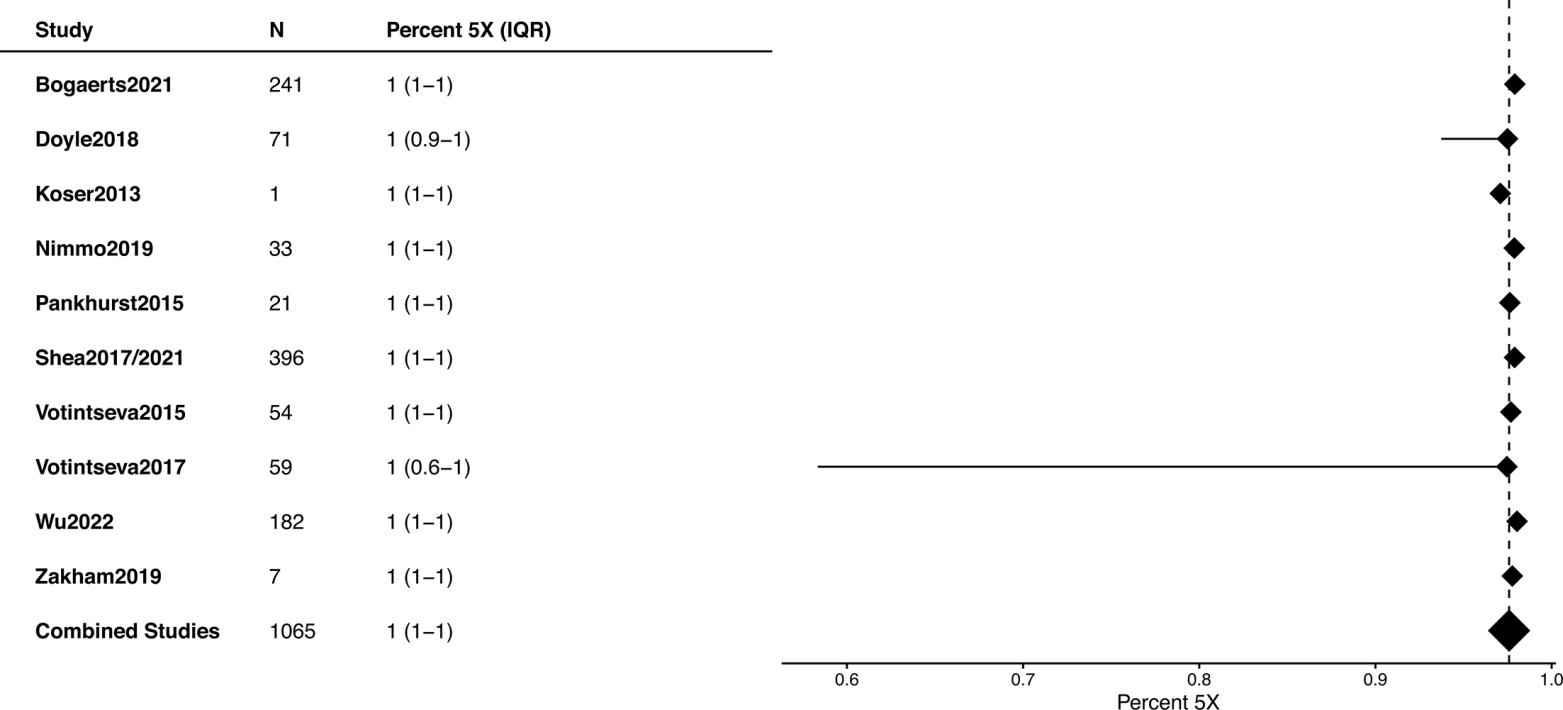
Distribution of percentage of sites represented by a minimum depth of 5 times (5×), for each study included in the meta-analysis.

The median coverage for 5 million reads per sample varied between 65.1 and 194, with a pooled median of 175.8 (IQR, 72.9–188.9) (Figs 3 and S6). The proportion of sites with ≥5× varied between 65.1 and 194 (IQR, 72.9–188.9) (Fig. 4). One dataset [16] exhibited notably poor sequencing quality, with median coverage below 50×, mapped percentages ranging from 0 to 77% and an average mapping depth of ≥5× at only 5% of sites. Those samples did not pass QC.

Analysis of sequencing quality and laboratory protocols indicated that the number of sequencing cycles was positively associated with higher mapped percentages (coefficients±se: 0.136±0.058, *P*=0.008) and median coverage per 5 million reads (0.542±0.120, *P*<0.001). However, other parameters, including heat inactivation (MGIT culture volume, temperature and time) and samples processed using chemical lysis, column-based or chemical-based purification, were excluded from the analysis due to missing data or rank deficiencies in the full model. This highlights the critical role of sequencing cycle optimization in ensuring high-quality sequencing outputs. The complete description of the statistical methods and corresponding results is provided in File S1.

Using the MAGMA pipeline, we evaluated WGS data for all FASTQ files from CPC among the 11 studies with available data. This pipeline was specifically selected for its suitability in scenarios where the depth of coverage may be low (minimum 5×) and contamination levels may be high (>50%). The MAGMA pipeline incorporates QC measures, mapping against the *Mtb* H37Rv reference, and employs machine-learning-based variant filtering tools [11]. We observed that the published WGS data from CPC varied from 50× to 100× median depth of coverage and from 75 to 80% mapped reads. These results suggest that most of the studies had successfully produced high-quality genome sequencing with low levels of contamination (sequence reads originating from non-*Mtb* DNA). This pipeline was specifically selected for its suitability in scenarios where a sample’s depth of coverage may be below 20× and/or contamination levels may exceed 50% [11,12].

In general, once FASTQ files are generated and undergo bioinformatic analyses, the genomic depth of coverage is a crucial metric for assessing WGS quality. It reflects how many times, on average, each base in the genome is sequenced. Three main factors influence the depth of coverage: number of sequences, mapping percentage and insert size. The number of sequence reads is primarily determined by the user’s specifications and the chosen sequencing platform. However, in rare cases where limited DNA is available for library preparation, the pre-library steps can limit the number of library sequences loaded onto the sequencing machine. Among the studies analysed, Votintseva *et al.*’s [20] FASTQ reports demonstrated the highest variability compared to others (Fig. 4).

The mapping percentage refers to the proportion of reads successfully mapped to the *Mtb* reference genome. It depends on the level of the target (non-contaminating) reads and the stringency of the mapping algorithm (e.g. MAGMA pipeline). Notably, pre-library steps such as saline pre-treatment on CPC samples influence the mapping percentage through effective contaminant removal methods. Insert size ideally should be at least twice the paired-end read length to avoid losing sequencing data due to read overlap. Pre-library steps, such as fragmentation, directly impact insert size. Several other factors influence the depth of coverage, but they are generally independent of pre-library steps. These include duplication levels (unless PCR cycles were adjusted to compensate for low input DNA), base calling qualities and unintended sequencing of adapter sequences due to missing inserts.

By understanding these factors and their interplay, researchers can optimize pre-library and sequencing processes to achieve the desired depth of coverage for accurate and reliable WGS data analysis. In our analysis, we observed that the number of sequencing-by-synthesis cycles and the read length determined by the Illumina kit used (e.g. 2×150, 2×250 or 2×300) impacted the mapped read percentage or adjusted coverage per 5 million reads analysis. Longer sequencing runs generally produced higher mapping accuracy and more uniform coverage, particularly in GC-rich regions of the *Mtb* genome.

In contrast, all studies reported a similar number of PCR amplification cycles (typically 15) during library preparation, and therefore no variation was available to assess their effect on duplication rates or amplification bias. It is well established, however, that excessive PCR amplification can lead to duplicate reads and reduced data quality if not properly controlled [36]. Because of incomplete metadata reporting, further correlation with other laboratory variables, such as culture volume, heat-inactivation conditions, pre-treatment, DNA extraction method, library QC and sequencing instrument, could not be explored.

Our study is limited by heterogeneity in the reporting of laboratory methods, incomplete metadata and the inability to fully separate CPC from subculture datasets in some BioProjects. Additionally, ONT data were scarce and not systematically assessed. Future work should address these gaps in prospective, standardized studies.

### Standardized workflow for WGS from primary liquid cultures

**Fig. 5.**
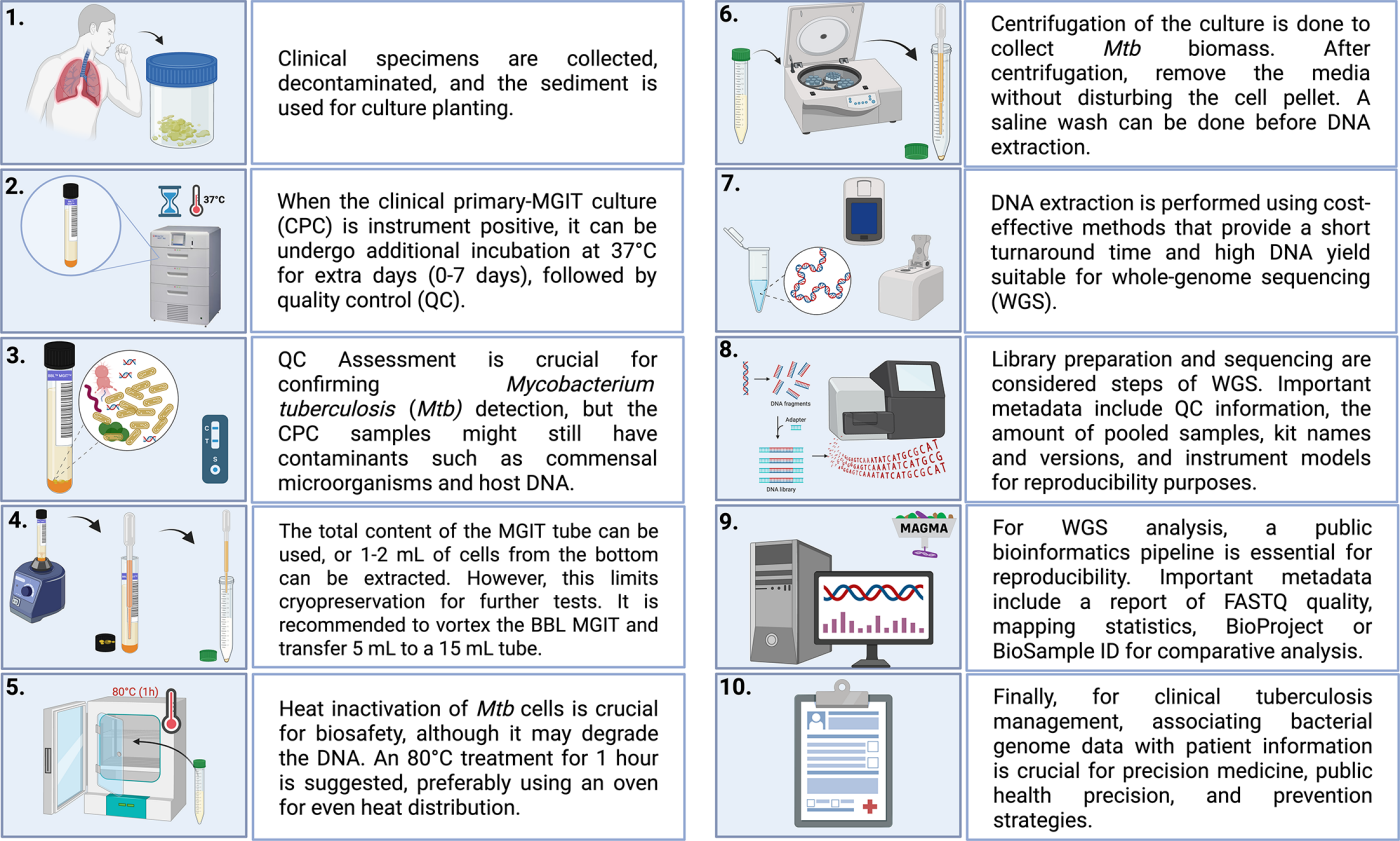
Proposed integration of WGS from *Mtb* clinical primary-MGIT cultures (CPCs) into routine care, with data retention for QC and future comparative studies. Created in BioRender. Warren, R. (2025) https://BioRender.com/wtrc0qi.

A comprehensive review of the existing literature has enabled us to propose potential standardization methods for WGS on CPCs (Fig. 5). In line with the approach proposed by Bustin *et al.* [37], our approach includes several key steps: sample heat inactivation, pre-treatment, DNA isolation, library preparation, sequencing and QC measures. These steps are designed to optimize CPC and achieve the desired results (Table 2).

**Table 2.** Suggested standardized reporting of WGS methods from clinical primary liquid MGIT cultures

Item to check	Importance	Item to check	Importance
Time of MGIT culture incubation at 37 °C	M	DNA concentration input to start the genomic library preparation	M
Volume of MGIT culture used for DNA extraction	M	Library preparation kit used. If performed modifications, specify it	M
MGIT culture heat-inactivation temperature	O	Genomic library QC based on dsDNA concentration (ng µl^−1^)	O
MGIT culture heat-inactivation time	O	Genomic library QC based on fragment size (bp)	O
If performed a pre-treatment prior to DNA extraction, specify	M	Cartridge version or capacity	M
Detailed DNA extraction method	M	Number of samples batched per run	M
DNA QC based on purity assessed by ratios 280/260 and 280/230	O	WGS sequencing instrument	M
DNA QC based on dsDNA concentration (ng µl^−1^)	M	Provide the fastq files BioProject or BioSample ID	M
DNA QC based on DNA integrity number	O	State if the fastq files were filtered before publishing	M

M: Minimum information; O: optimal information.

In this workflow, a 500 µl aliquot of the decontaminated sputum sediment is used for MGIT culture planting. When the culture is reported as positive, the isolate can undergo further incubation at 37 °C until a large enough batch of samples is available for processing, ideally up to one extra week (7 days). The batch of samples depends on the sequencing technology and flow cell capacity. For all positive cultures, the presence of *Mtb* should be confirmed by the immunochromatographic assay antigen test, or smear microscopy should be performed to confirm the presence of acid-fast bacilli as QC steps. Alternatively, *Mtb* presence may be verified through rapid molecular diagnostic assays such as GeneXpert or Line Probe Assay. Next, the MGIT culture is vortexed and a 5 ml aliquot of the homogenized culture is collected. This is important, as it allows conducting additional tests, such as drug susceptibility testing on the remaining culture and cryopreservation.

The aliquot is heat-inactivated to mitigate the occupational hazard of *Mtb* infection and allow one to handle samples outside the BSL3 containment facility. Even though most included studies used 95 °C for 15 to 120 min, we propose heat inactivation at 80 °C for 60 min because high temperatures for an extended period can lead to DNA degradation and lyse the *Mtb* cells. The heat-inactivated aliquot is then centrifuged to collect the *Mtb* biomass, removing the culture media without disturbing the cell pellet.

A saline wash with Tween, Triton X, DNAse 1X or TE can be performed before DNA extraction to assist in removing contaminant cell depletion. DNA extraction and QC can then be performed, ideally using a cost-effective method with a short turnaround time. Next, genomic library preparation, QC and sequencing are performed, and raw sequencing data are analysed using a bioinformatics pipeline that produces reproducible outputs.

### Minimal information for standardized reporting of laboratory methods

To facilitate interlaboratory data comparison, we propose a set of minimum information that should be reported when publishing studies on WGS from CPC samples, similarly proposed by Bustin *et al.* [37] on real-time qPCR.

Important metadata includes QC information, the amount of pooled samples, kit names and versions and instrument models for reproducibility purposes. Important metadata includes a report of FASTQ quality, mapping statistics and BioProject or BioSample ID for comparative analysis. Finally, the *Mtb* genomic data and patient information are integrated and communicated for clinical tuberculosis management, public health and prevention.

## Conclusions

While there have been significant advancements in the WGS application on *Mtb* CPC, several critical areas need standardization and further exploration to enhance the efficiency and reliability of WGS applications for early detection of *Mtb*. This systematic review and meta-analysis demonstrates that WGS from MGIT primary cultures is highly feasible, with >95% of datasets meeting QC thresholds. Importantly, we show that sequencing quality is influenced primarily by sequencing cycles, rather than heat inactivation, extraction or purification methods. We observed the use of alternative methods of DNA extraction instead of CTAB, which could significantly accelerate the adoption of WGS in CPC samples within the diagnostics settings, leading to improved patient care.

Replacing EPC with CPC terminology better reflects laboratory practice (including re-incubation and batching) while emphasizing the advantage of avoiding subculture, which prolongs turnaround times and risks loss of heteroresistance detection. Standardizing CPC workflows and reporting could accelerate clinical implementation of WGS in high-burden, resource-limited settings, improving patient management and TB control programmes.

Lastly, we have identified a promising potential in evaluating and optimizing the application of ONT library preparation kits on CPC samples, even when starting with low DNA inputs. This optimization could further enhance the efficiency and accuracy of the ONT WGS process in CPCs, which could favour sequencing decentralization. The implementation of these strategies could lead to improved patient care and expedited diagnosis of drug resistant TB.

## Supplementary material

10.1099/mgen.0.001565 Uncited Supplementary Material 1.

10.1099/mgen.0.001565 Uncited Table S1.

10.1099/mgen.0.001565 Uncited Table S2.

## Abbreviations

CPC: clinical primary culture
EPC: early positive culture
GI: growth index
IQR: interquartile range
*Mtb*: *Mycobacterium tuberculosis*
NTM: non-tuberculous mycobacteria
ONT: Oxford Nanopore Technology
QC: quality control
qPCR: quantitative PCR
WGS: whole-genome sequencing
