# Supplementary material for: Mycobacterium tuberculosis cultured in MGIT media for whole-genome sequencing application: a systematic literature review and meta-analysis
Source: Microb Genom. 2025 Nov 24;11(11):001565. doi: 10.1099/mgen.0.001565 (PMC12645293; doi:10.1099/mgen.0.001565)
Supplement: Uncited Supplementary Material 1. [file mgen-11-01565-s001.pdf]

Supplementary Figure 1. The selection of studies employing a Whole-genome sequencing approach on primary liquid Mycobacteria Growth Indicator Tube (MGIT) cultures of *Mycobacterium tuberculosis.*

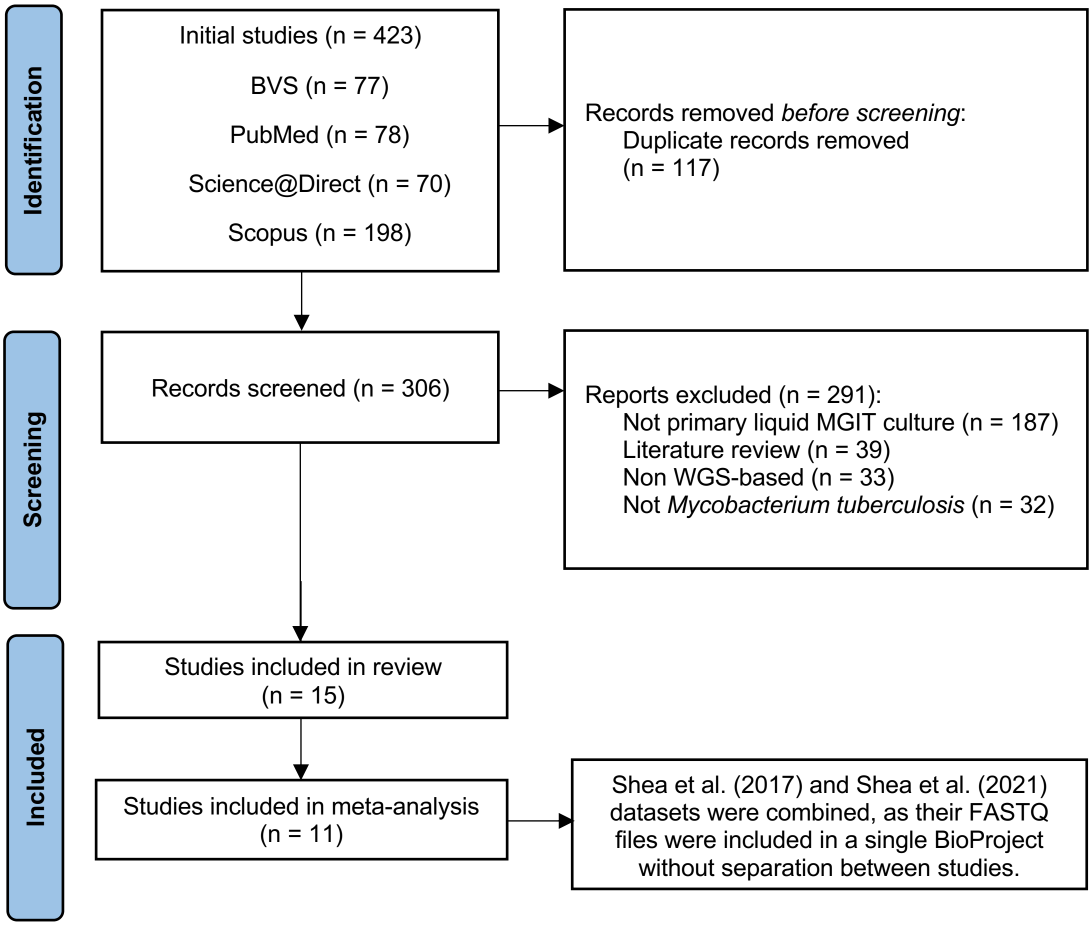

Supplementary Figure 2. Box plot showing the distribution of median genome coverage across studies included in the meta-analysis.

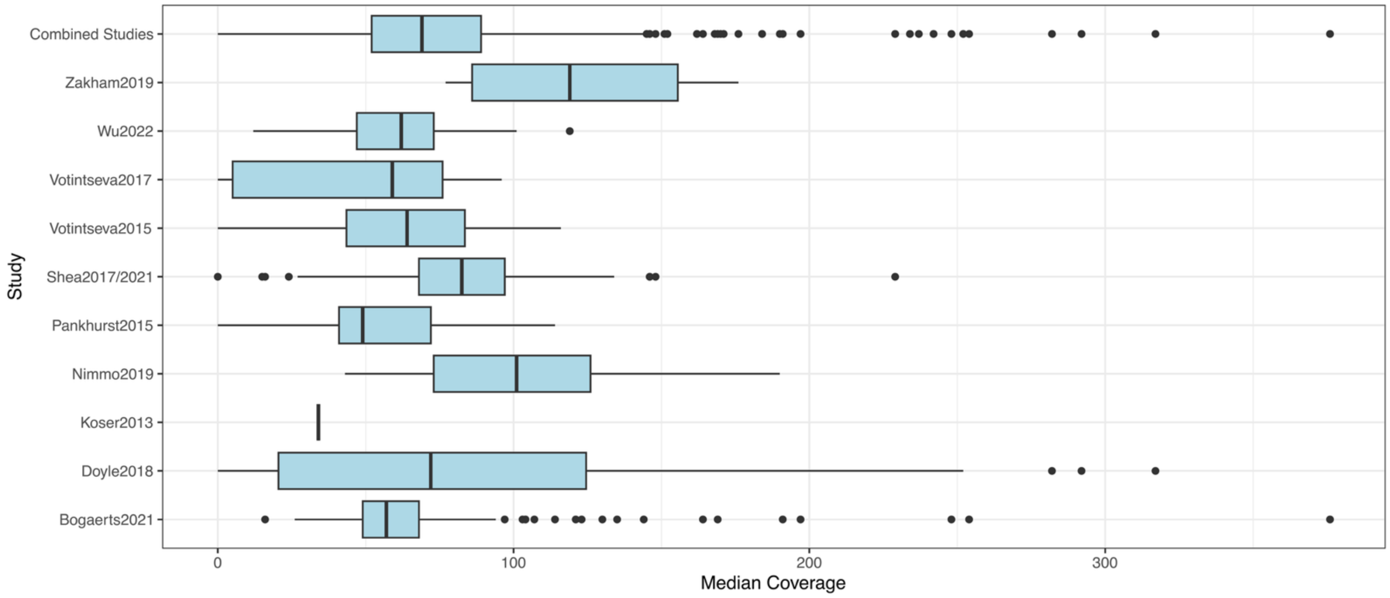

Supplementary Figure 3. QQ plot showing the distribution of median genome coverage across studies included in the meta-analysis.

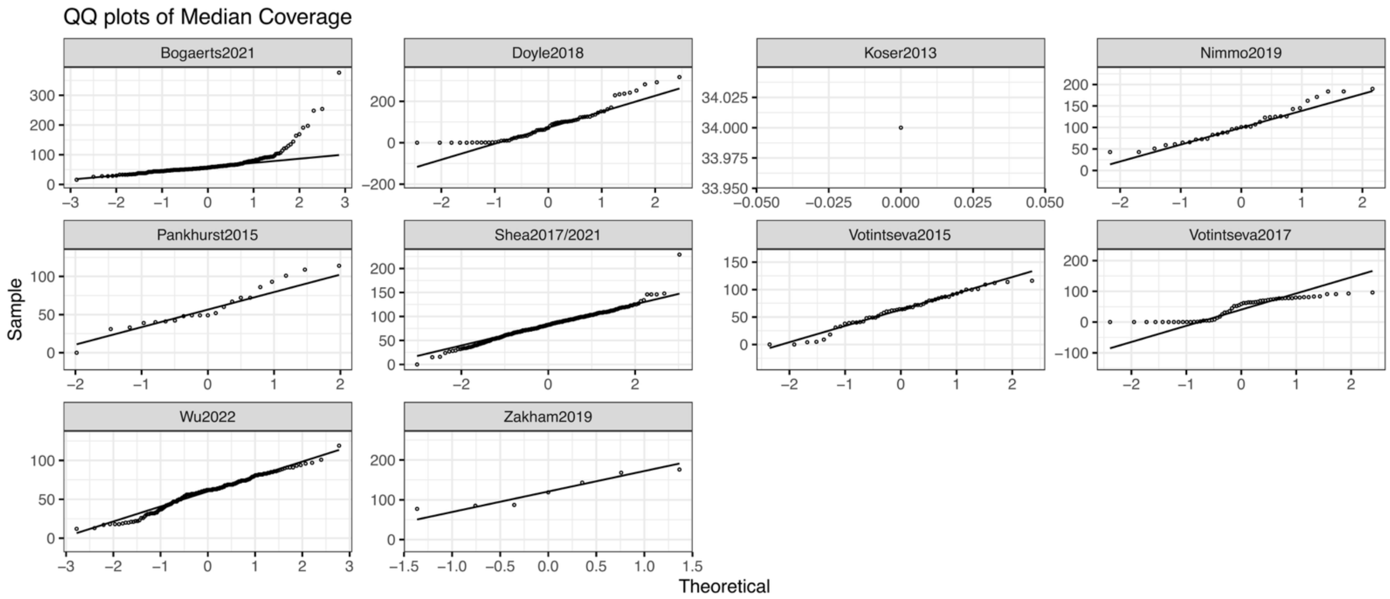

Supplementary Figure 4. Box plot showing the distribution of mapped percentage across studies included in the meta-analysis considering the reference *Mycobacterium tuberculosis* H37Rv.

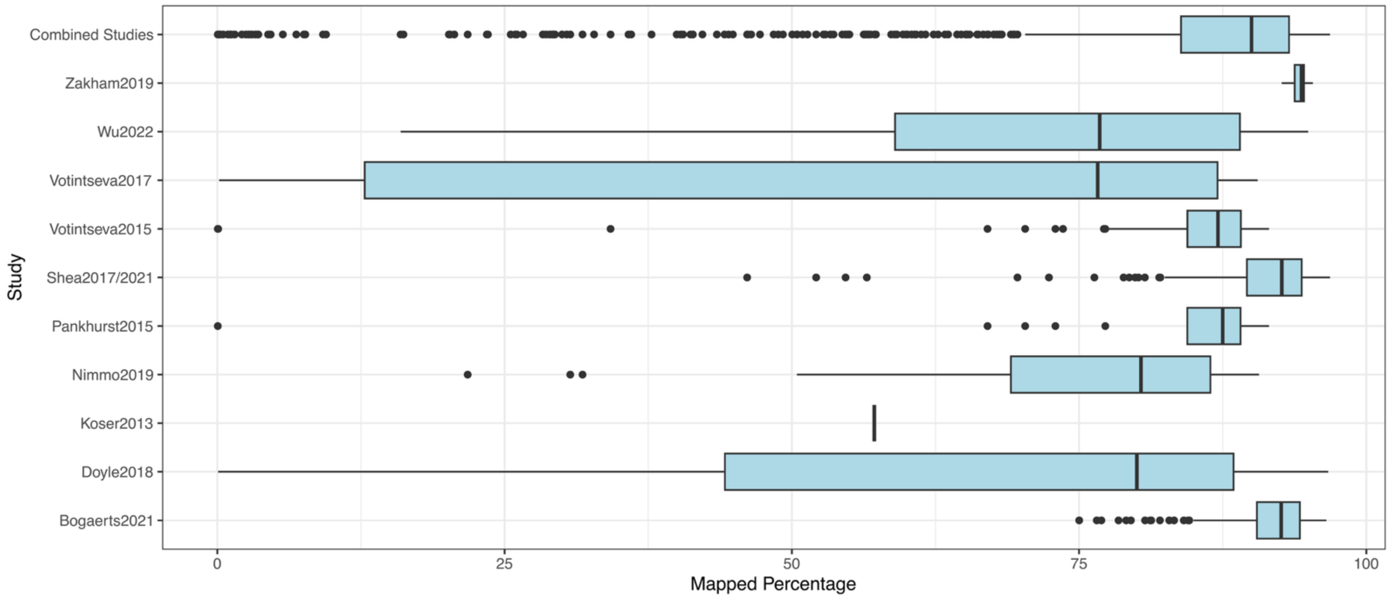

Supplementary Figure 5. QQ plot showing the distribution of mapped percentage coverage across studies included in the meta-analysis considering the reference *Mycobacterium tuberculosis* H37Rv.

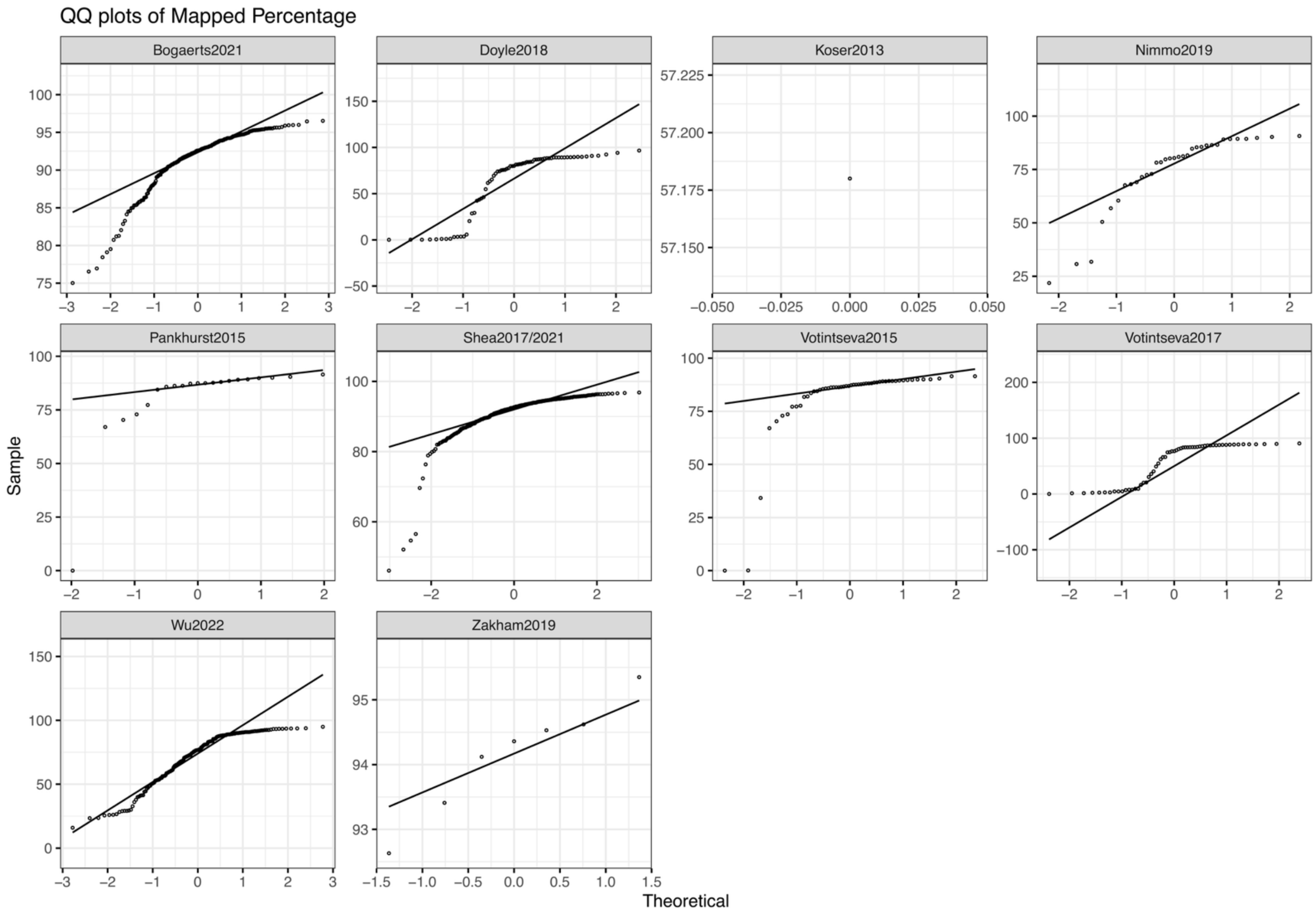

Supplementary Figure 6. Box plot showing the distribution of adjusted coverage per five million reads.

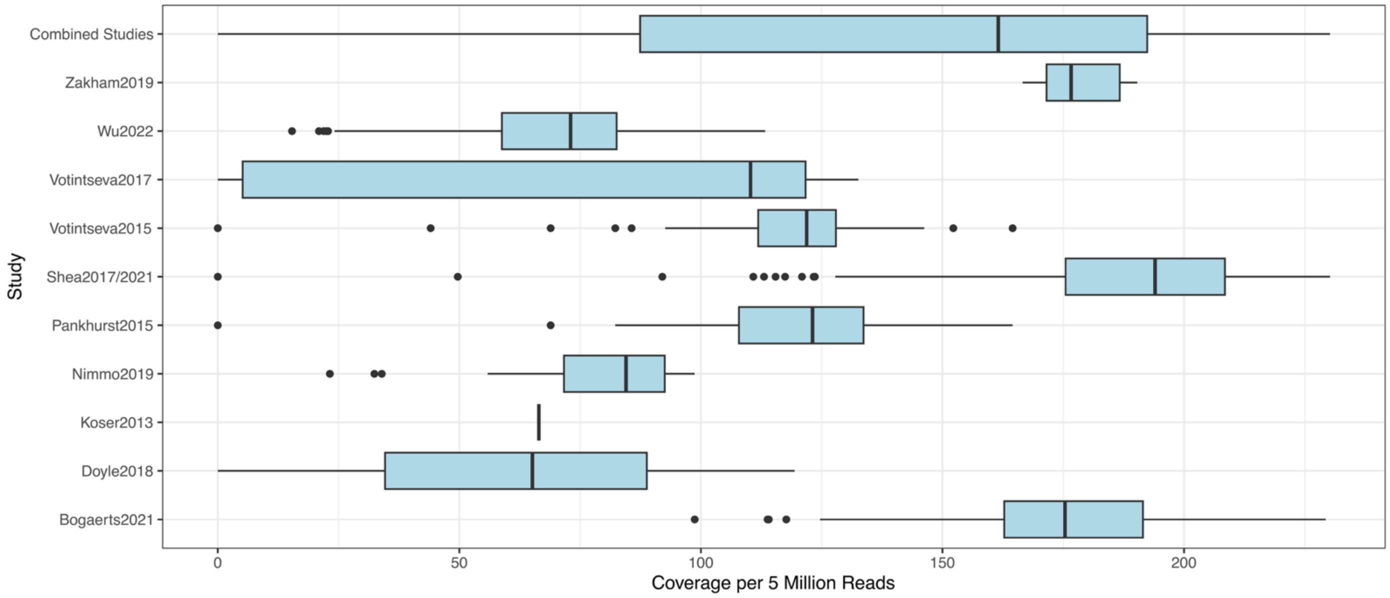

Supplementary Material 1. Detailed Statistical Approaches and Results Applied to the Meta-Analysis.

EPC Review

Gian van der Spuy

10 October 2025

Median Coverage

```
Linear mixed model fit by REML. t-tests use Satterthwaite's method [
lmerModLmerTest]
Formula: median_coverage ~ heat_inactivation_volume + enzymatic_lysis +
    mechanical_lysis + purification_bead_cleanup + library_prep_kit +
    sequencing_cycles + (1 | study)
   Data: df

REML criterion at convergence: 10533.6

Scaled residuals:
    Min      1Q  Median      3Q     Max
-2.4914 -0.4881 -0.0533  0.4395  9.0098

Random effects:
 Groups   Name        Variance Std.Dev.
 study    (Intercept)  424.6   20.61
 Residual             1190.3   34.50
Number of obs: 1065, groups:  study, 10

Fixed effects:
                                Estimate Std. Error        df t value Pr(>|t|)
(Intercept)                     77.23256  109.54832   3.61618   0.705    0.524
heat_inactivation_volume.L     -50.33101   37.31763   7.10729  -1.349    0.219
heat_inactivation_volume.Q     -24.57331   28.01873   4.20458  -0.877    0.428
enzymatic_lysisYES              56.98933   32.00255   2.67335   1.781    0.184
mechanical_lysisYES             16.68486   29.27669   1.87252   0.570    0.630
purification_bead_cleanupYES   -25.48901   45.23307  10.63522  -0.564    0.585
library_prep_kitNextera DNA XT -54.55757   54.15427   5.47587  -1.007    0.356
```

```
sequencing_cycles               0.08482    0.11997   1.90051   0.707    0.556

Correlation of Fixed Effects:
            (Intr) ht__.L ht__.Q en_YES mc_YES p__YES l__NDX
ht_nctvt_.L -0.712
ht_nctvt_.Q -0.518  0.687
enzymtc_YES -0.302 -0.352 -0.271
mchncl_lYES -0.690  0.277  0.213  0.458
prfctn__YES -0.630  0.676  0.520  0.000  0.000
lbr__NDNAXT -0.904  0.715  0.550  0.248  0.541  0.659
sqncng_cycl -0.618  0.455  0.170  0.000  0.609  0.000  0.329
```

```
                                        2.5 %     97.5 %
.sig01                             5.22678267  17.150162
.sigma                            33.05697284  36.000005
(Intercept)                      -66.51885715 221.518658
heat_inactivation_volume.L      -107.15290990   6.250918
heat_inactivation_volume.Q       -62.83257970  13.810965
enzymatic_lysisYES                18.75873997  95.219909
mechanical_lysisYES              -13.58785191  46.957582
purification_bead_cleanupYES     -98.19751103  47.219488
library_prep_kitNextera DNA XT -133.08404327  23.968907
sequencing_cycles                 -0.04056228   0.209202
```

Backward Elimination Model

```
Linear mixed model fit by REML. t-tests use Satterthwaite's method [
lmerModLmerTest]
Formula: median_coverage ~ (1 | study)
   Data: df

REML criterion at convergence: 10588.9

Scaled residuals:
    Min      1Q  Median      3Q     Max
-2.5024 -0.4865 -0.0519  0.4407  9.0046

Random effects:
 Groups   Name        Variance Std.Dev.
 study    (Intercept)  490.4   22.15
 Residual             1191.0   34.51
Number of obs: 1065, groups:  study, 10
```

```
Fixed effects:
            Estimate Std. Error     df t value Pr(>|t|)
(Intercept)   73.821      7.501  7.345   9.842 1.72e-05 ***
---
Signif. codes:  0 '***' 0.001 '**' 0.01 '*' 0.05 '.' 0.1 ' ' 1

               2.5 %   97.5 %
.sig01      12.57060 37.30583
.sigma      33.09149 36.04154
(Intercept) 58.26956 89.30071
```

Study	N	Median Coverage (IQR)
**Bogaerts2021**	241	57 (49−68)
**Doyle2018**	71	72 (20.5−124.5)
**Koser2013**	1	34 (34−34)
**Nimmo2019**	33	101 (73−126)
**Pankhurst2015**	21	49 (41−72)
**Shea2017/2021**	396	82.5 (68−97)
**Votintseva2015**	54	64 (43.5−83.5)
**Votintseva2017**	59	59 (5−76)
**Wu2022**	182	62 (47−73)
**Zakham2019**	7	119 (86−155.5)
**Combined Studies**	1065	65.2 (62.8−81.8)

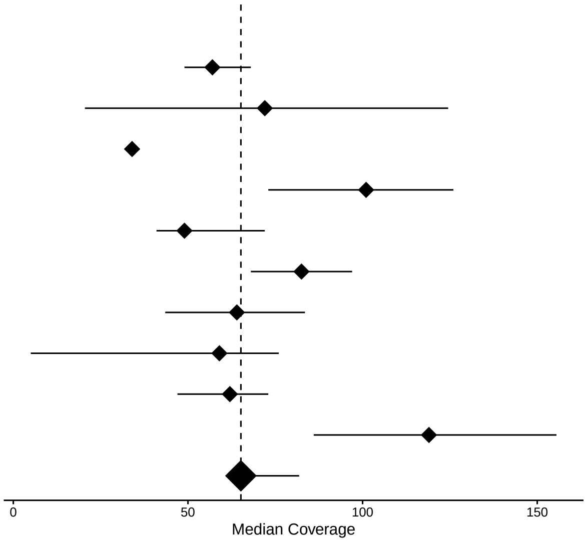

Mapped Percentage

```
Linear mixed model fit by REML. t-tests use Satterthwaite's method [
lmerModLmerTest]
Formula: mapped_percentage ~ heat_inactivation_volume + enzymatic_lysis +
    mechanical_lysis + purification_bead_cleanup + library_prep_kit +
    sequencing_cycles + (1 | study)
   Data: df

REML criterion at convergence: 8932.4

Scaled residuals:
```

```
    Min      1Q  Median      3Q     Max 
-5.0169 -0.1340  0.1121  0.2855  2.0913 

Random effects:
 Groups   Name        Variance Std.Dev.
 study    (Intercept) 163.1    12.77   
 Residual             261.4    16.17   
Number of obs: 1065, groups:  study, 10

Fixed effects:
                               Estimate Std. Error       df t value Pr(>|t|)
(Intercept)                     63.6959    63.5896   2.8613   1.002    0.394
heat_inactivation_volume.L      -9.1126    20.5503   4.5590  -0.443    0.678
heat_inactivation_volume.Q     -16.4264    16.0569   3.1609  -1.023    0.378
enzymatic_lysisYES               2.6086    19.0970   2.3621   0.137    0.902
mechanical_lysisYES             -0.3231    18.1110   1.9109  -0.018    0.987
purification_bead_cleanupYES   -14.4962    24.2716   6.1571  -0.597    0.572
library_prep_kitNextera DNA XT -17.5967    30.3909   3.7866  -0.579    0.595
sequencing_cycles                0.1204     0.0741   1.9272   1.625    0.250

Correlation of Fixed Effects:
            (Intr) ht__.L ht__.Q en_YES mc_YES p__YES l__NDX
ht_nctvt_.L -0.683                                          
ht_nctvt_.Q -0.469  0.641                                   
enzymtc_YES -0.325 -0.361 -0.267                            
mchncl_lYES -0.736  0.311  0.230  0.475                     
prfctn__YES -0.565  0.602  0.445  0.000  0.000              
lbr__NDNAXT -0.893  0.666  0.492  0.283  0.596  0.576       
sqncng_cycl -0.659  0.510  0.185  0.000  0.610  0.000  0.364


                                      2.5 %      97.5 %
.sig01                           3.52759306  10.4092661
.sigma                          15.49334152  16.8728581
(Intercept)                    -13.40474004 140.6482775
heat_inactivation_volume.L     -37.58656741  19.4288437
heat_inactivation_volume.Q     -36.56104989   3.6736561
enzymatic_lysisYES             -18.88789975  24.1051323
mechanical_lysisYES            -18.87089844  18.2247448
purification_bead_cleanupYES   -50.35052439  21.3582167
library_prep_kitNextera DNA XT -57.79614631  22.6027106
sequencing_cycles                0.04435639   0.1967426
```

Backward Elimination Model

```
Linear mixed model fit by REML. t-tests use Satterthwaite's method [
lmerModLmerTest]
Formula: mapped_percentage ~ sequencing_cycles + (1 | study)
   Data: df

REML criterion at convergence: 8975.2

Scaled residuals:
    Min      1Q  Median      3Q     Max
-4.9967 -0.1362  0.1122  0.2973  2.0525

Random effects:
 Groups   Name        Variance Std.Dev.
 study    (Intercept)  64.52    8.032
 Residual             261.30   16.165
Number of obs: 1065, groups:  study, 10

Fixed effects:
                  Estimate Std. Error       df t value Pr(>|t|)
(Intercept)       38.67895   11.23481  7.45186   3.443  0.00980 **
sequencing_cycles  0.10674    0.03001  7.43606   3.556  0.00839 **
---
Signif. codes:  0 '***' 0.001 '**' 0.01 '*' 0.05 '.' 0.1 ' ' 1

Correlation of Fixed Effects:
            (Intr)
sqncng_cycl -0.969


                        2.5 %     97.5 %
.sig01             4.34285725 12.5239828
.sigma            15.49895537 16.8793354
(Intercept)       16.82464184 60.2615801
sequencing_cycles  0.04905335  0.1650965
```

Study	N	Mapped Percentage (IQR)
Bogaerts2021	241	92.6 (90.5–94.2)
Doyle2018	71	80 (44.2–88.4)
Koser2013	1	57.2 (57.2–57.2)
Nimmo2019	33	80.4 (69.1–86.4)
Pankhurst2015	21	87.5 (84.4–89)
Shea2017/2021	396	92.6 (89.6–94.4)
Votintseva2015	54	87.1 (84.4–89.1)
Votintseva2017	59	76.6 (12.8–87)
Wu2022	182	76.8 (59–89)
Zakham2019	7	94.4 (93.8–94.6)
Combined Studies	1065	91.2 (71.7–91.2)

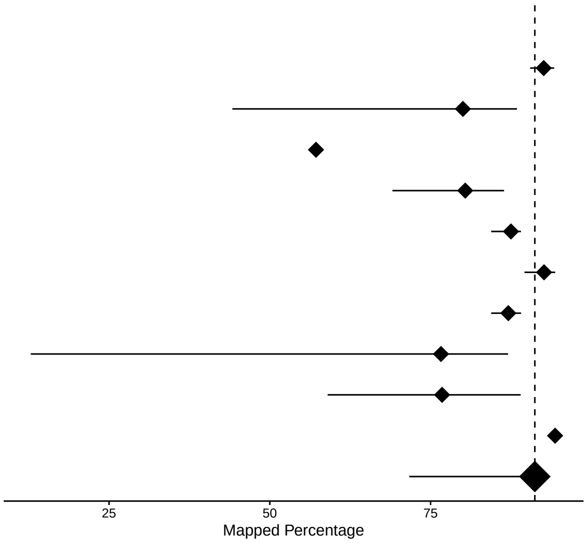

Mean Insert Size

```
Linear mixed model fit by REML. t-tests use Satterthwaite's method [
lmerModLmerTest]
Formula: avg_insert_size ~ heat_inactivation_volume + enzymatic_lysis +
    mechanical_lysis + purification_bead_cleanup + library_prep_kit +
    sequencing_cycles + (1 | study)
   Data: df

REML criterion at convergence: 12014.4

Scaled residuals:
    Min      1Q  Median      3Q     Max
-4.0616 -0.5151 -0.1005  0.6368  5.3057

Random effects:
 Groups   Name        Variance Std.Dev.
 study    (Intercept) 5118     71.54
 Residual             4822     69.44
Number of obs: 1065, groups:  study, 10

Fixed effects:
                                Estimate Std. Error       df t value Pr(>|t|)
(Intercept)                    -253.2936   342.1552   2.5032  -0.740    0.522
```

```
heat_inactivation_volume.L       43.5368   106.3208    3.4102   0.409    0.707
heat_inactivation_volume.Q      -14.7840    85.6319    2.6688  -0.173    0.875
enzymatic_lysisYES                7.9607   104.6137    2.2201   0.076    0.946
mechanical_lysisYES              87.4345   101.3278    1.9541   0.863    0.481
purification_bead_cleanupYES     14.0319   122.8157    4.2157   0.114    0.914
library_prep_kitNextera DNA XT  139.4315   159.5953    3.0061   0.874    0.446
sequencing_cycles                 1.0441     0.4142    1.9639   2.521    0.130

Correlation of Fixed Effects:
            (Intr) ht__.L ht__.Q en_YES mc_YES p__YES l__NDX
ht_nctvt_.L -0.662
ht_nctvt_.Q -0.436  0.607
enzymtc_YES -0.341 -0.369 -0.265
mchncl_lYES -0.765  0.337  0.241  0.485
prfctn__YES -0.517  0.538  0.386  0.000  0.000
lbr__NDNAXT -0.885  0.628  0.450  0.308  0.635  0.507
sqncng_cycl -0.685  0.551  0.196  0.000  0.611  0.000  0.388


                                      2.5 %     97.5 %
.sig01                           21.3032486  57.745584
.sigma                           66.5452492  72.470077
(Intercept)                    -645.8823550 138.474569
heat_inactivation_volume.L      -92.2745204 179.714657
heat_inactivation_volume.Q     -115.5332864  85.775779
enzymatic_lysisYES             -105.9979504 121.919333
mechanical_lysisYES             -16.5679009 191.436944
purification_bead_cleanupYES   -152.7236653 180.787395
library_prep_kitNextera DNA XT  -56.4638047 335.326729
sequencing_cycles                 0.6188457   1.471183
```

Backward Elimination Model

```
Linear mixed model fit by REML. t-tests use Satterthwaite's method [
lmerModLmerTest]
Formula: avg_insert_size ~ sequencing_cycles + (1 | study)
   Data: df

REML criterion at convergence: 12079.8

Scaled residuals:
    Min      1Q  Median      3Q     Max
-4.0563 -0.5180 -0.1072  0.6352  5.3045
```

```
Random effects:
 Groups   Name        Variance Std.Dev.
 study    (Intercept) 2997     54.74
 Residual             4819     69.42
Number of obs: 1065, groups:  study, 10

Fixed effects:
                  Estimate Std. Error      df t value Pr(>|t|)
(Intercept)         7.6040    73.7346  8.1648   0.103  0.92035
sequencing_cycles   0.8223     0.1971  8.1147   4.172  0.00302 **
---
Signif. codes:  0 '***' 0.001 '**' 0.01 '*' 0.05 '.' 0.1 ' ' 1

Correlation of Fixed Effects:
            (Intr)
sqncng_cycl -0.969


                         2.5 %     97.5 %
.sig01              32.0076705  83.341945
.sigma              66.5577660  72.485147
(Intercept)       -134.8339400 150.711143
sequencing_cycles    0.4394093   1.203005
```

Study	N	Mean Insert Size (IQR)
**Bogaerts2021**	241	375.5 (342.5−428.7)
**Doyle2018**	71	178.6 (169.7−193.3)
**Koser2013**	1	246.1 (246.1−246.1)
**Nimmo2019**	33	194.5 (180.6−211.5)
**Pankhurst2015**	21	315.5 (267.4−351.9)
**Shea2017/2021**	396	485.8 (424−526.8)
**Votintseva2015**	54	328.4 (288.1−360.3)
**Votintseva2017**	59	342.5 (269.6−364.8)
**Wu2022**	182	209.2 (199.8−222.8)
**Zakham2019**	7	388.4 (371.6−416)
**Combined Studies**	1065	385.4 (232.1−472.8)

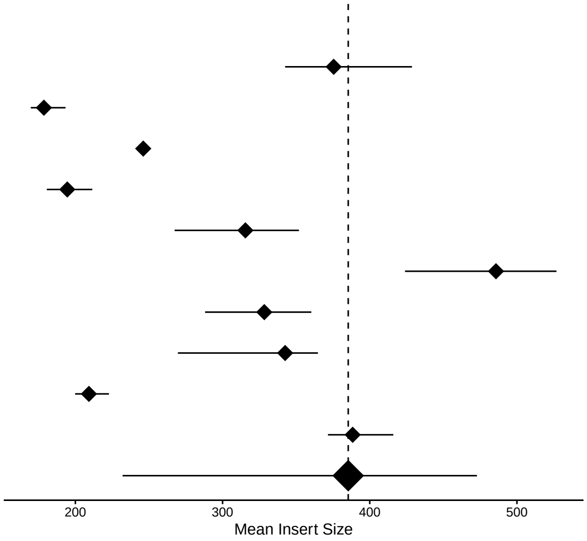

Adjusted Coverage per 5 Million Reads

```
Linear mixed model fit by REML. t-tests use Satterthwaite's method [
lmerModLmerTest]
Formula: adjusted_coverage_per_5_million_reads ~ heat_inactivation_volume +
    enzymatic_lysis + mechanical_lysis + purification_bead_cleanup +
    library_prep_kit + sequencing_cycles + (1 | study)
   Data: df

REML criterion at convergence: 10103.6

Scaled residuals:
    Min      1Q  Median      3Q     Max
-6.7162 -0.4617  0.1488  0.6333  2.1319

Random effects:
 Groups   Name        Variance Std.Dev.
 study    (Intercept) 807.8    28.42
 Residual             790.9    28.12
Number of obs: 1065, groups:  study, 10

Fixed effects:
                               Estimate Std. Error       df t value Pr(>|t|)
(Intercept)                    -80.3406   136.2522   2.5177  -0.590   0.6041
heat_inactivation_volume.L      -3.6861    42.4429   3.4638  -0.087   0.9356
heat_inactivation_volume.Q     -29.9554    34.1186   2.6900  -0.878   0.4513
enzymatic_lysisYES               2.7632    41.6147   2.2235   0.066   0.9526
mechanical_lysisYES             13.0750    40.2590   1.9476   0.325   0.7769
purification_bead_cleanupYES    -2.7567    49.0999   4.3072  -0.056   0.9577
library_prep_kitNextera DNA XT   1.2482    63.6491   3.0417   0.020   0.9856
sequencing_cycles                0.5346     0.1646   1.9578   3.248   0.0855 .
---
Signif. codes:  0 '***' 0.001 '**' 0.01 '*' 0.05 '.' 0.1 ' ' 1

Correlation of Fixed Effects:
            (Intr) ht__.L ht__.Q en_YES mc_YES p__YES l__NDX
ht_nctvt_.L -0.663
ht_nctvt_.Q -0.438  0.609
enzymtc_YES -0.340 -0.369 -0.265
mchncl_lYES -0.763  0.335  0.241  0.484
prfctn__YES -0.520  0.542  0.390  0.000  0.000
lbr__NDNAXT -0.886  0.630  0.453  0.306  0.633  0.512
```

```
sqncng_cycl -0.684  0.548  0.195  0.000  0.611  0.000  0.387
```

```
                                      2.5 %     97.5 %
.sig01                             8.371407 22.9163916
.sigma                            26.951281 29.3509627
(Intercept)                     -236.922876 75.9337058
heat_inactivation_volume.L       -58.140445 50.9061103
heat_inactivation_volume.Q       -70.191195 10.2092470
enzymatic_lysisYES               -42.562996 48.0894422
mechanical_lysisYES              -28.140892 54.2909577
purification_bead_cleanupYES     -69.762453 64.2490650
library_prep_kitNextera DNA XT   -77.151774 79.6481988
sequencing_cycles                  0.365998  0.7038299
```

Backward Elimination Model

```
Linear mixed model fit by REML. t-tests use Satterthwaite's method [
lmerModLmerTest]
Formula: adjusted_coverage_per_5_million_reads ~ sequencing_cycles + (1 |
    study)
   Data: df

REML criterion at convergence: 10156.8

Scaled residuals:
    Min      1Q  Median      3Q     Max
-6.7164 -0.4633  0.1510  0.6321  2.1198

Random effects:
 Groups   Name        Variance Std.Dev.
 study    (Intercept) 397.1    19.93
 Residual             790.6    28.12
Number of obs: 1065, groups:  study, 10

Fixed effects:
                   Estimate Std. Error        df t value Pr(>|t|)
(Intercept)       -63.01686   27.03661   7.87967  -2.331 0.048574 *
sequencing_cycles   0.48862    0.07226   7.84536   6.762 0.000157 ***
---
Signif. codes:  0 '***' 0.001 '**' 0.01 '*' 0.05 '.' 0.1 ' ' 1

Correlation of Fixed Effects:
```

```
            (Intr)
sqncng_cycl -0.969

                        2.5 %      97.5 %
.sig01             11.4071590  30.5314926
.sigma             26.9583255  29.3593585
(Intercept)      -115.4170487 -10.7963225
sequencing_cycles   0.3489083   0.6285712
```

Study	N	Adjusted Coverage per 5 Million Reads
**Bogaerts2021**	241	175.4 (162.8−191.5)
**Doyle2018**	71	65.1 (34.6−88.8)
**Koser2013**	1	66.4 (66.4−66.4)
**Nimmo2019**	33	84.5 (71.7−92.5)
**Pankhurst2015**	21	123.1 (107.9−133.7)
**Shea2017/2021**	396	194 (175.5−208.5)
**Votintseva2015**	54	121.9 (111.9−128)
**Votintseva2017**	59	110.3 (5.1−121.7)
**Wu2022**	182	73 (58.8−82.5)
**Zakham2019**	7	176.6 (171.5−186.7)
**Combined Studies**	1065	175.8 (72.9−188.9)

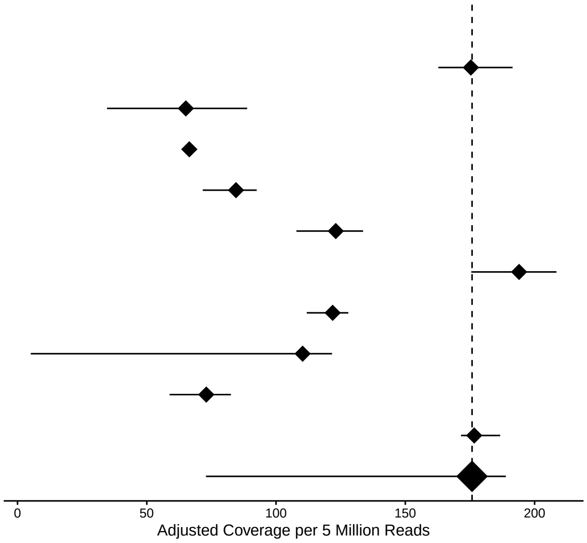

Percentage 5x Coverage

```
Linear mixed model fit by REML. t-tests use Satterthwaite's method [
lmerModLmerTest]
Formula:
pct_5x ~ heat_inactivation_volume + enzymatic_lysis + mechanical_lysis +
    purification_bead_cleanup + library_prep_kit + sequencing_cycles +
    (1 | study)
   Data: df

REML criterion at convergence: -1015.9

Scaled residuals:
    Min      1Q  Median      3Q     Max
-6.5870 -0.0041  0.0178  0.0445  1.7759
```

```
Random effects:
 Groups   Name        Variance Std.Dev.
 study    (Intercept) 0.01004  0.1002
 Residual             0.02138  0.1462
Number of obs: 1065, groups:  study, 10

Fixed effects:
                                 Estimate Std. Error        df t value
(Intercept)                     0.6824163  0.5140347  3.1661338   1.328
heat_inactivation_volume.L      0.1084120  0.1704137  5.5851539   0.636
heat_inactivation_volume.Q     -0.0312882  0.1305899  3.5836728  -0.240
enzymatic_lysisYES              0.0008936  0.1524048  2.4831848   0.006
mechanical_lysisYES            -0.0014560  0.1422204  1.8830874  -0.010
purification_bead_cleanupYES   -0.0095022  0.2039206  7.9443553  -0.047
library_prep_kitNextera DNA XT -0.0077511  0.2496812  4.4700024  -0.031
sequencing_cycles               0.0008032  0.0005823  1.9045090   1.379
                               Pr(>|t|)
(Intercept)                       0.272
heat_inactivation_volume.L        0.550
heat_inactivation_volume.Q        0.824
enzymatic_lysisYES                0.996
mechanical_lysisYES               0.993
purification_bead_cleanupYES      0.964
library_prep_kitNextera DNA XT    0.977
sequencing_cycles                 0.307

Correlation of Fixed Effects:
            (Intr) ht__.L ht__.Q en_YES mc_YES p__YES l__NDX
ht_nctvt_.L -0.697
ht_nctvt_.Q -0.493  0.664
enzymtc_YES -0.314 -0.357 -0.269
mchncl_lYES -0.714  0.295  0.222  0.467
prfctn__YES -0.597  0.639  0.482  0.000  0.000
lbr__NDNAXT -0.898  0.690  0.520  0.266  0.570  0.617
sqncng_cycl -0.640  0.483  0.178  0.000  0.610  0.000  0.347


                                      2.5 %     97.5 %
.sig01                          0.0260419363 0.08211917
.sigma                          0.1401208403 0.15259759
(Intercept)                     0.0361901050 1.32744737
heat_inactivation_volume.L     -0.1386023882 0.35596772
```

```
heat_inactivation_volume.Q     -0.2017539904 0.13889814
enzymatic_lysisYES             -0.1746925928 0.17647983
mechanical_lysisYES            -0.1468213043 0.14390928
purification_bead_cleanupYES   -0.3240400755 0.30503574
library_prep_kitNextera DNA XT -0.3528888225 0.33738659
sequencing_cycles               0.0002060311 0.00140267
```

Backward Elimination Model

```
Linear mixed model fit by REML. t-tests use Satterthwaite's method [
lmerModLmerTest]
Formula: pct_5x ~ (1 | study)
   Data: df

REML criterion at convergence: -1039.6

Scaled residuals:
    Min      1Q  Median      3Q     Max
-6.5870  0.0012  0.0222  0.0490  1.7422

Random effects:
 Groups   Name        Variance Std.Dev.
 study    (Intercept) 0.008278 0.09098
 Residual             0.021366 0.14617
Number of obs: 1065, groups:  study, 10

Fixed effects:
            Estimate Std. Error      df t value Pr(>|t|)
(Intercept)  0.91874    0.03089 9.14365   29.74 2.06e-10 ***
---
Signif. codes:  0 '***' 0.001 '**' 0.01 '*' 0.05 '.' 0.1 ' ' 1


                 2.5 %    97.5 %
.sig01      0.05613496 0.1483329
.sigma      0.14014719 0.1526268
(Intercept) 0.85593015 0.9827388
```

Study	N	Percent 5X (IQR)
**Bogaerts2021**	241	1 (1−1)
**Doyle2018**	71	1 (0.9−1)
**Koser2013**	1	1 (1−1)
**Nimmo2019**	33	1 (1−1)
**Pankhurst2015**	21	1 (1−1)
**Shea2017/2021**	396	1 (1−1)
**Votintseva2015**	54	1 (1−1)
**Votintseva2017**	59	1 (0.6−1)
**Wu2022**	182	1 (1−1)
**Zakham2019**	7	1 (1−1)
**Combined Studies**	1065	1 (1−1)

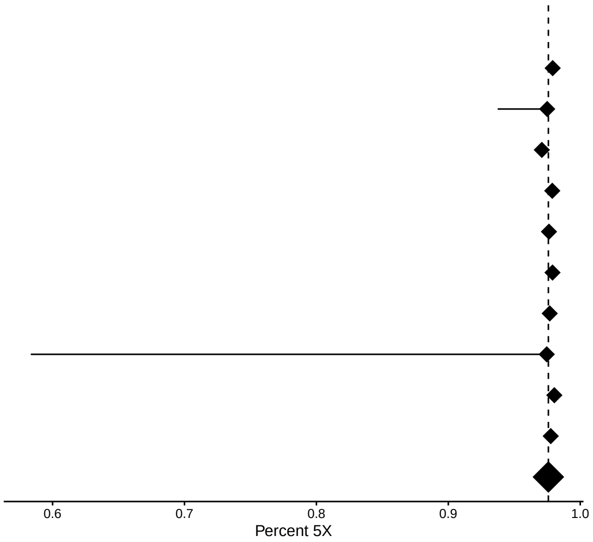

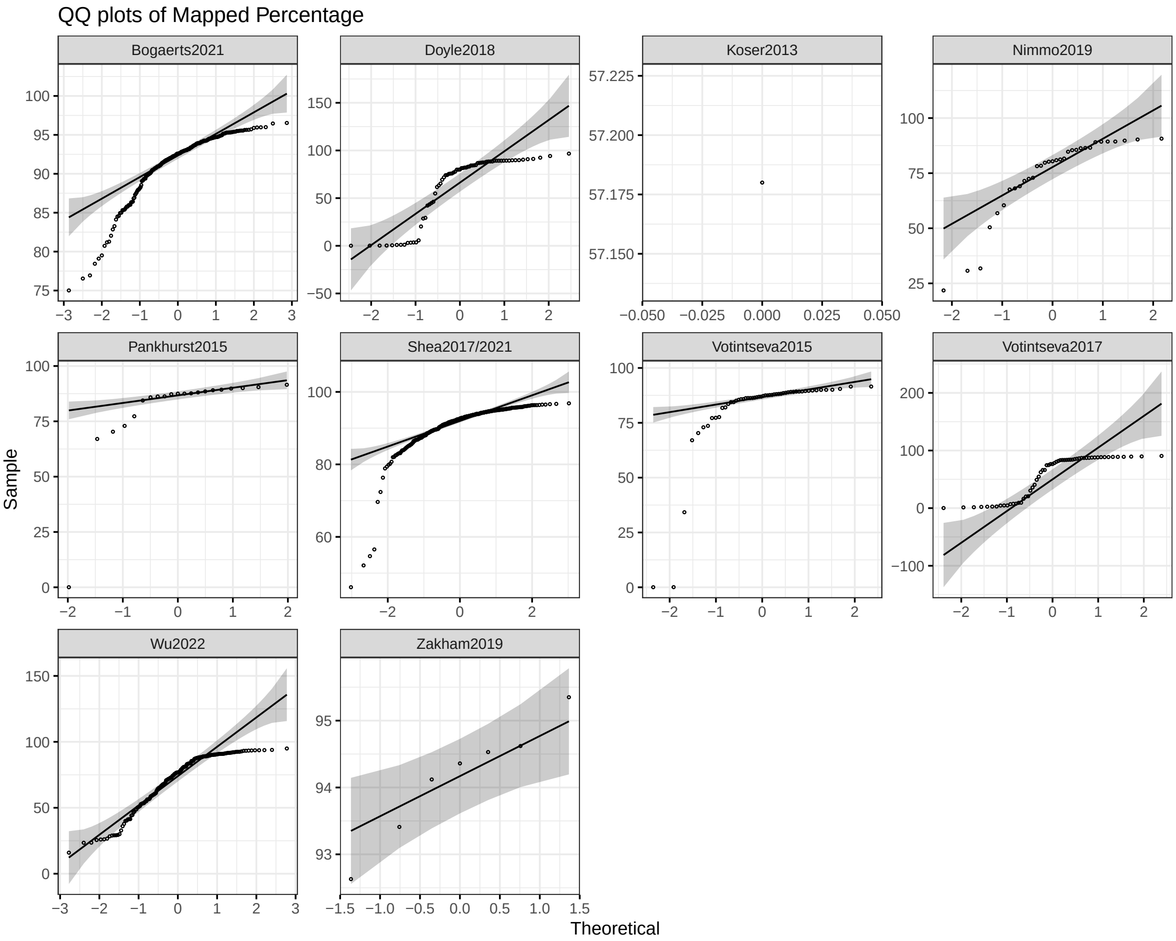

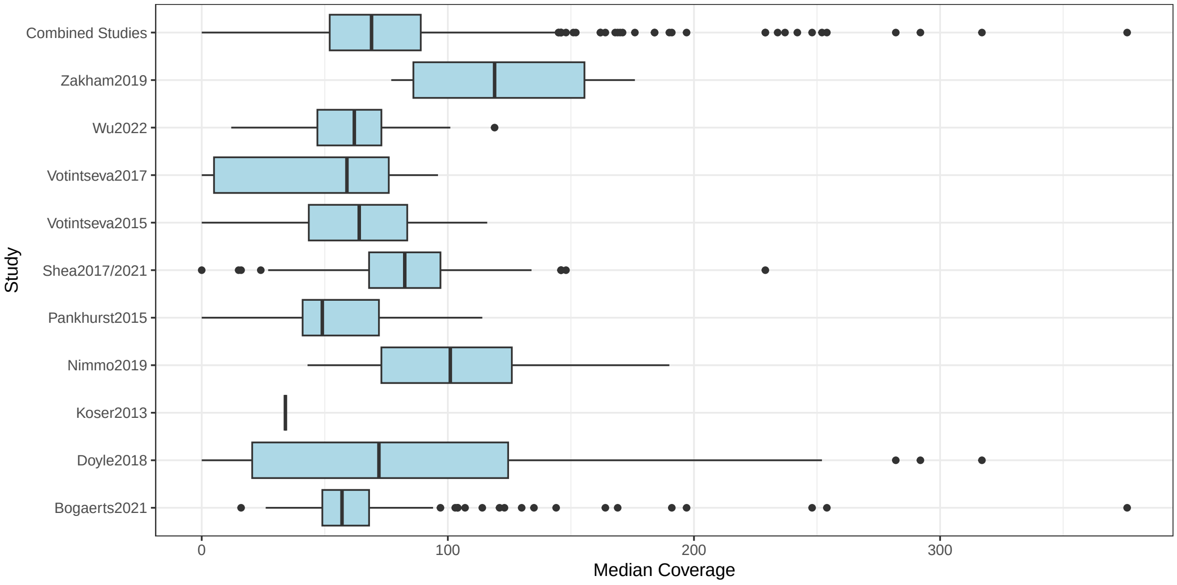
Combined Studies
Zakham2019
Wu2022
Votintseva2017
Votintseva2015
Shea2017/2021
Pankhurst2015
Nimmo2019
Koser2013
Doyle2018
Bogaerts2021
Study
0
100
200
300
Median Coverage

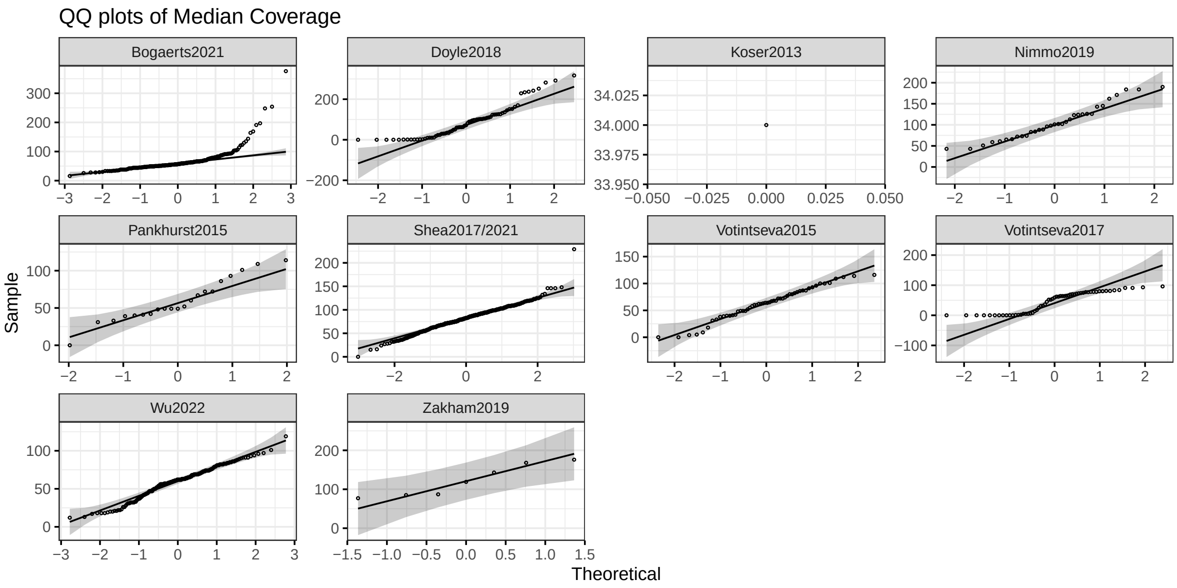
QQ plots of Median Coverage
Bogaerts2021
Doyle2018
Koser2013
Nimmo2019
Pankhurst2015
Shea2017/2021
Votintseva2015
Votintseva2017
Wu2022
Zakham2019
Sample
Theoretical

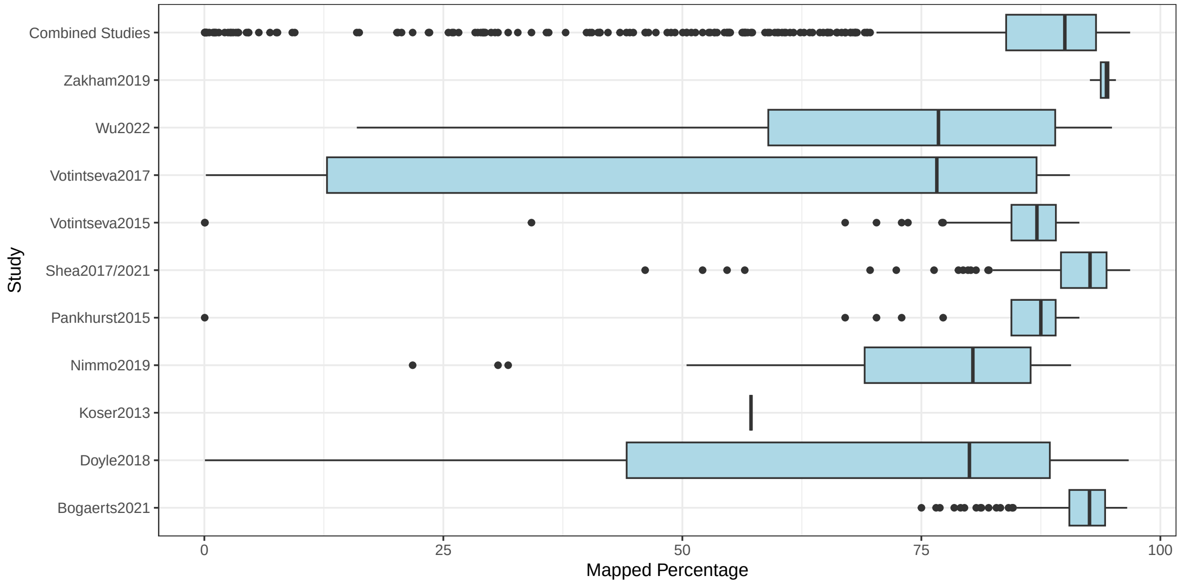
Combined Studies
Zakham2019
Wu2022
Votintseva2017
Votintseva2015
Shea2017/2021
Pankhurst2015
Nimmo2019
Koser2013
Doyle2018
Bogaerts2021
Study
0
25
50
75
100
Mapped Percentage

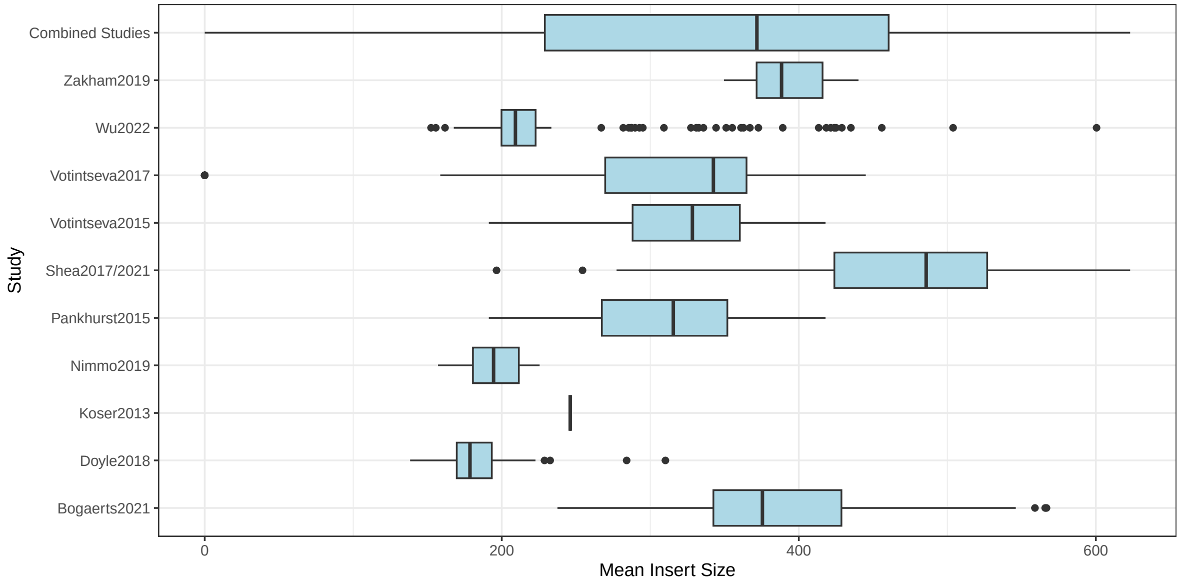
Combined Studies
Zakham2019
Wu2022
Votintseva2017
Votintseva2015
Shea2017/2021
Pankhurst2015
Nimmo2019
Koser2013
Doyle2018
Bogaerts2021
Study
0
200
400
600
Mean Insert Size

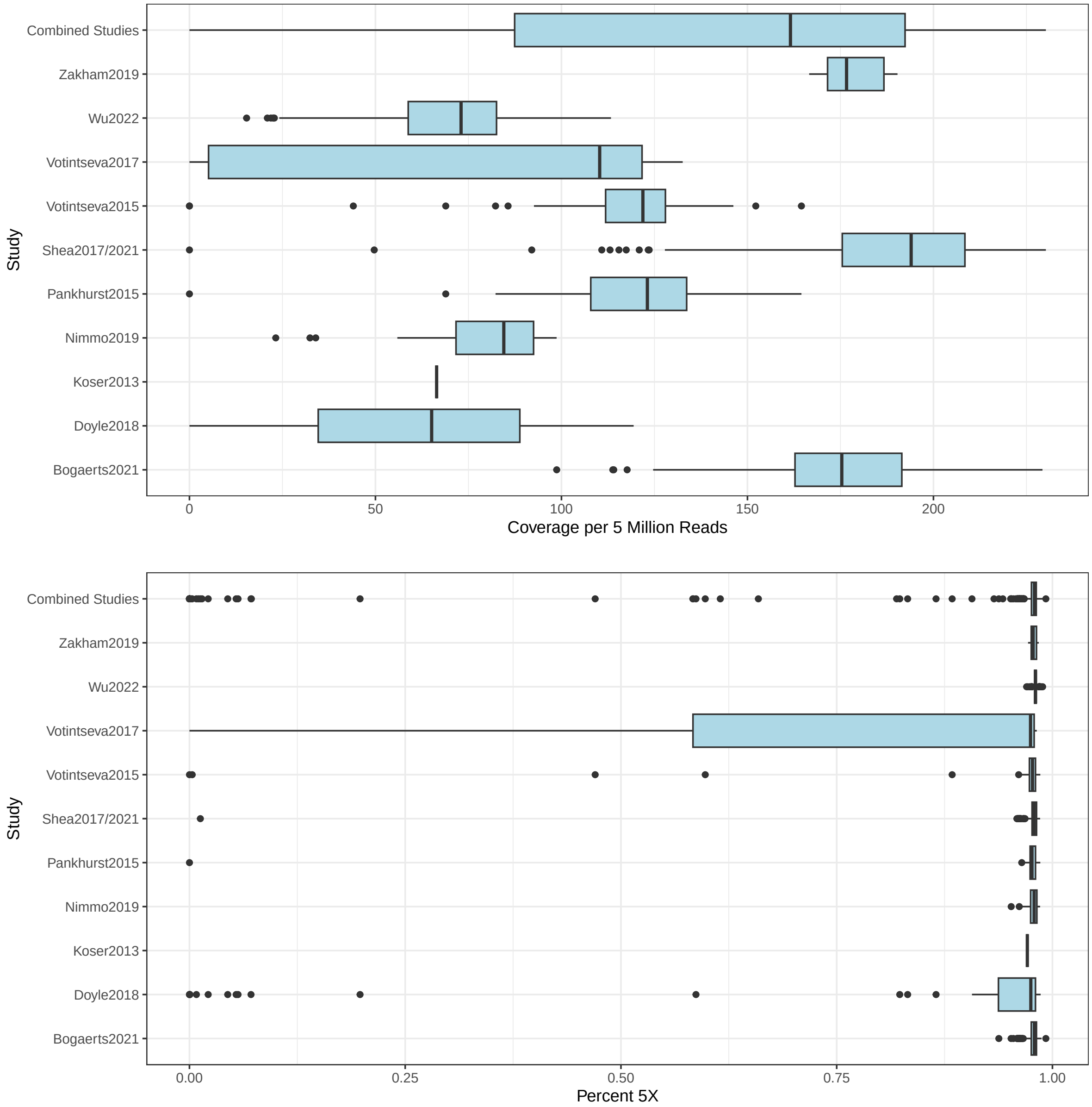
Combined Studies
Zakham2019
Wu2022
Votintseva2017
Votintseva2015
Shea2017/2021
Pankhurst2015
Nimmo2019
Koser2013
Doyle2018
Bogaerts2021
Study
0
50
100
150
200
Coverage per 5 Million Reads
Combined Studies
Zakham2019
Wu2022
Votintseva2017
Votintseva2015
Shea2017/2021
Pankhurst2015
Nimmo2019
Koser2013
Doyle2018
Bogaerts2021
Study
0.00
0.25
0.50
0.75
1.00
Percent 5X

Analysis

Gian van der Spuy

10 October 2025

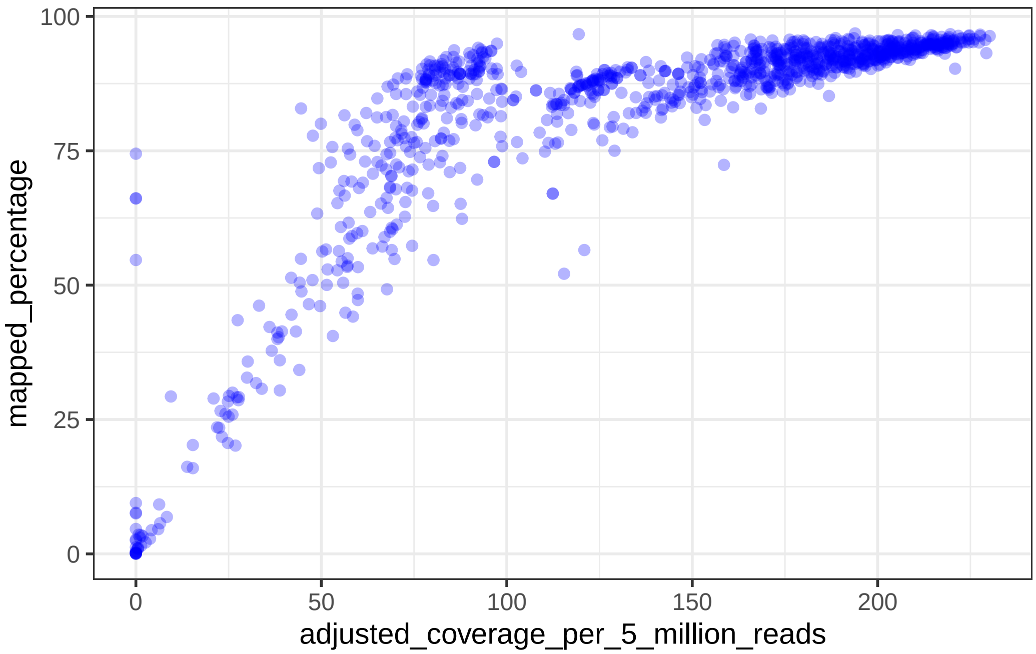

Mapped Percentage

All Variables

```
Linear mixed model fit by REML. t-tests use Satterthwaite's method [
lmerModLmerTest]
Formula: mapped_percentage ~ heat_inactivation_volume + enzymatic_lysis +
    mechanical_lysis + purification_bead_cleanup + library_prep_kit +
    sequencing_cycles + (1 | study)
```

```
   Data: df

REML criterion at convergence: 8932.4

Scaled residuals:
    Min      1Q  Median      3Q     Max
-5.0169 -0.1340  0.1121  0.2855  2.0913

Random effects:
 Groups   Name        Variance Std.Dev.
 study    (Intercept) 163.1    12.77
 Residual             261.4    16.17
Number of obs: 1065, groups:  study, 10

Fixed effects:
                               Estimate Std. Error       df t value Pr(>|t|)
(Intercept)                     63.6959    63.5896   2.8613   1.002    0.394
heat_inactivation_volume.L      -9.1126    20.5503   4.5590  -0.443    0.678
heat_inactivation_volume.Q     -16.4264    16.0569   3.1609  -1.023    0.378
enzymatic_lysisYES               2.6086    19.0970   2.3621   0.137    0.902
mechanical_lysisYES             -0.3231    18.1110   1.9109  -0.018    0.987
purification_bead_cleanupYES   -14.4962    24.2716   6.1571  -0.597    0.572
library_prep_kitNextera DNA XT -17.5967    30.3909   3.7866  -0.579    0.595
sequencing_cycles                0.1204     0.0741   1.9272   1.625    0.250

Correlation of Fixed Effects:
            (Intr) ht__.L ht__.Q en_YES mc_YES p__YES l__NDX
ht_nctvt_.L -0.683
ht_nctvt_.Q -0.469  0.641
enzymtc_YES -0.325 -0.361 -0.267
mchncl_lYES -0.736  0.311  0.230  0.475
prfctn__YES -0.565  0.602  0.445  0.000  0.000
lbr__NDNAXT -0.893  0.666  0.492  0.283  0.596  0.576
sqncng_cycl -0.659  0.510  0.185  0.000  0.610  0.000  0.364

                                      2.5 %      97.5 %
.sig01                           3.52759306  10.4092661
.sigma                          15.49334152  16.8728581
(Intercept)                    -13.40474004 140.6482775
heat_inactivation_volume.L     -37.58656741  19.4288437
heat_inactivation_volume.Q     -36.56104989   3.6736561
enzymatic_lysisYES             -18.88789975  24.1051323
```

```
mechanical_lysisYES            -18.87089844  18.2247448
purification_bead_cleanupYES   -50.35052439  21.3582167
library_prep_kitNextera DNA XT -57.79614631  22.6027106
sequencing_cycles                0.04435639   0.1967426
```

Pooled Estimate

```
     25%      50%      75% 
71.67615 91.21402 91.21402 
```

Lysis Method

```
Linear mixed model fit by REML. t-tests use Satterthwaite's method [
lmerModLmerTest]
Formula: mapped_percentage ~ enzymatic_lysis + mechanical_lysis + (1 |
    study)
   Data: df

REML criterion at convergence: 8964.7

Scaled residuals: 
    Min      1Q  Median      3Q     Max 
-5.0329 -0.1322  0.1171  0.2889  2.0802 

Random effects:
 Groups   Name        Variance Std.Dev.
 study    (Intercept) 182.2    13.50   
 Residual             261.5    16.17   
Number of obs: 1065, groups:  study, 10

Fixed effects:
                    Estimate Std. Error      df t value Pr(>|t|)    
(Intercept)           86.354     10.727   6.053   8.050 0.000188 ***
enzymatic_lysisYES    -6.685     11.064   6.262  -0.604 0.566948    
mechanical_lysisYES  -10.592     11.036   6.183  -0.960 0.373178    
---
Signif. codes:  0 '***' 0.001 '**' 0.01 '*' 0.05 '.' 0.1 ' ' 1

Correlation of Fixed Effects:
            (Intr) en_YES
enzymtc_YES -0.714       
mchncl_lYES -0.854  0.529
```

```
                          2.5 %     97.5 %
.sig01                 7.117437  19.295212
.sigma                15.503798  16.885008
(Intercept)           67.004272 105.651489
enzymatic_lysisYES   -26.627535  13.218134
mechanical_lysisYES -30.444172   9.314143
```

Purification Bead-Cleanup

```
Linear mixed model fit by REML. t-tests use Satterthwaite's method [
lmerModLmerTest]
Formula: mapped_percentage ~ purification_bead_cleanup + (1 | study)
   Data: df

REML criterion at convergence: 8971.3

Scaled residuals:
    Min      1Q  Median      3Q     Max
-5.0366 -0.1327  0.1184  0.2885  2.0769

Random effects:
 Groups   Name        Variance Std.Dev.
 study    (Intercept) 180.2    13.42
 Residual             261.4    16.17
Number of obs: 1065, groups:  study, 10

Fixed effects:
                             Estimate Std. Error     df t value Pr(>|t|)
(Intercept)                    71.676     13.476  6.734   5.319  0.00125 **
purification_bead_cleanupYES    6.188     14.282  6.827   0.433  0.67819
---
Signif. codes:  0 '***' 0.001 '**' 0.01 '*' 0.05 '.' 0.1 ' ' 1

Correlation of Fixed Effects:
            (Intr)
prfctn__YES -0.944


                                  2.5 %   97.5 %
.sig01                         7.668408 20.53865
.sigma                        15.502627 16.88370
(Intercept)                   45.586458 97.76585
purification_bead_cleanupYES -21.525240 33.74709
```

Sequencing Cycles

```
Linear mixed model fit by REML. t-tests use Satterthwaite's method [
lmerModLmerTest]
Formula: mapped_percentage ~ sequencing_cycles + (1 | study)
   Data: df

REML criterion at convergence: 8975.2

Scaled residuals:
    Min      1Q  Median      3Q     Max
-4.9967 -0.1362  0.1122  0.2973  2.0525

Random effects:
 Groups   Name        Variance Std.Dev.
 study    (Intercept)  64.52    8.032
 Residual             261.30   16.165
Number of obs: 1065, groups:  study, 10

Fixed effects:
                  Estimate Std. Error       df t value Pr(>|t|)
(Intercept)       38.67895   11.23481  7.45186   3.443  0.00980 **
sequencing_cycles  0.10674    0.03001  7.43606   3.556  0.00839 **
---
Signif. codes:  0 '***' 0.001 '**' 0.01 '*' 0.05 '.' 0.1 ' ' 1

Correlation of Fixed Effects:
            (Intr)
sqncng_cycl -0.969


                        2.5 %     97.5 %
.sig01             4.34285725 12.5239828
.sigma            15.49895537 16.8793354
(Intercept)       16.82464184 60.2615801
sequencing_cycles  0.04905335  0.1650965
```

Adjusted Coverage for 5 Million Reads

All Variables

```
Linear mixed model fit by REML. t-tests use Satterthwaite's method [
```

```
lmerModLmerTest]
Formula: adjusted_coverage_per_5_million_reads ~ heat_inactivation_volume +
    enzymatic_lysis + mechanical_lysis + purification_bead_cleanup +
    library_prep_kit + sequencing_cycles + (1 | study)
   Data: df

REML criterion at convergence: 10103.6

Scaled residuals:
    Min      1Q  Median      3Q     Max
-6.7162 -0.4617  0.1488  0.6333  2.1319

Random effects:
 Groups   Name        Variance Std.Dev.
 study    (Intercept) 807.8    28.42
 Residual             790.9    28.12
Number of obs: 1065, groups:  study, 10

Fixed effects:
                               Estimate Std. Error       df t value Pr(>|t|)
(Intercept)                    -80.3406   136.2522   2.5177  -0.590   0.6041
heat_inactivation_volume.L      -3.6861    42.4429   3.4638  -0.087   0.9356
heat_inactivation_volume.Q     -29.9554    34.1186   2.6900  -0.878   0.4513
enzymatic_lysisYES               2.7632    41.6147   2.2235   0.066   0.9526
mechanical_lysisYES             13.0750    40.2590   1.9476   0.325   0.7769
purification_bead_cleanupYES    -2.7567    49.0999   4.3072  -0.056   0.9577
library_prep_kitNextera DNA XT   1.2482    63.6491   3.0417   0.020   0.9856
sequencing_cycles                0.5346     0.1646   1.9578   3.248   0.0855 .
---
Signif. codes:  0 '***' 0.001 '**' 0.01 '*' 0.05 '.' 0.1 ' ' 1

Correlation of Fixed Effects:
            (Intr) ht__.L ht__.Q en_YES mc_YES p__YES l__NDX
ht_nctvt_.L -0.663
ht_nctvt_.Q -0.438  0.609
enzymtc_YES -0.340 -0.369 -0.265
mchncl_lYES -0.763  0.335  0.241  0.484
prfctn__YES -0.520  0.542  0.390  0.000  0.000
lbr__NDNAXT -0.886  0.630  0.453  0.306  0.633  0.512
sqncng_cycl -0.684  0.548  0.195  0.000  0.611  0.000  0.387

                                     2.5 %     97.5 %
```

```
.sig01                                8.371407 22.9163916
.sigma                               26.951281 29.3509627
(Intercept)                        -236.922876 75.9337058
heat_inactivation_volume.L          -58.140445 50.9061103
heat_inactivation_volume.Q          -70.191195 10.2092470
enzymatic_lysisYES                  -42.562996 48.0894422
mechanical_lysisYES                 -28.140892 54.2909577
purification_bead_cleanupYES        -69.762453 64.2490650
library_prep_kitNextera DNA XT      -77.151774 79.6481988
sequencing_cycles                     0.365998  0.7038299
```

```
     25%      50%      75%
 72.8756 175.8084 188.8834
```

Pooled Estimate

```
     25%      50%      75%
 72.8756 175.8084 188.8834
```

Lysis Method

```
Linear mixed model fit by REML. t-tests use Satterthwaite's method [
lmerModLmerTest]
Formula:
adjusted_coverage_per_5_million_reads ~ enzymatic_lysis + mechanical_lysis +
    (1 | study)
   Data: df

REML criterion at convergence: 10150.6

Scaled residuals:
    Min      1Q  Median      3Q     Max
-6.7142 -0.4613  0.1465  0.6343  2.1349

Random effects:
 Groups   Name        Variance Std.Dev.
 study    (Intercept) 2628.4   51.27
 Residual              790.9   28.12
Number of obs: 1065, groups:  study, 10

Fixed effects:
```

```
                    Estimate Std. Error      df t value Pr(>|t|)
(Intercept)          161.462     40.121   6.734   4.024  0.00545 **
enzymatic_lysisYES   -49.310     41.084   6.795  -1.200  0.27022
mechanical_lysisYES  -48.338     41.068   6.783  -1.177  0.27883
---
Signif. codes:  0 '***' 0.001 '**' 0.01 '*' 0.05 '.' 0.1 ' ' 1

Correlation of Fixed Effects:
            (Intr) en_YES
enzymtc_YES -0.749
mchncl_lYES -0.852  0.566
```

```
                         2.5 %    97.5 %
.sig01               28.48261  72.14777
.sigma               26.96493  29.36749
(Intercept)          88.80637 234.06032
enzymatic_lysisYES  -123.70142  25.02724
mechanical_lysisYES -122.64403  26.02984
```

Purification Bead-Cleanup

```
Linear mixed model fit by REML. t-tests use Satterthwaite's method [
lmerModLmerTest]
Formula: adjusted_coverage_per_5_million_reads ~ purification_bead_cleanup +
    (1 | study)
   Data: df

REML criterion at convergence: 10159.9

Scaled residuals:
    Min      1Q  Median      3Q     Max
-6.7143 -0.4592  0.1485  0.6345  2.1342

Random effects:
 Groups   Name        Variance Std.Dev.
 study    (Intercept) 2634.4   51.33
 Residual              790.9   28.12
Number of obs: 1065, groups:  study, 10

Fixed effects:
                             Estimate Std. Error     df t value Pr(>|t|)
(Intercept)                    69.203     51.369  7.559   1.347    0.217
```

```
purification_bead_cleanupYES   48.593     54.240  7.603   0.896    0.398

Correlation of Fixed Effects:
            (Intr)
prfctn__YES -0.947

                                 2.5 %    97.5 %
.sig01                        30.59635  77.20902
.sigma                        26.96500  29.36757
(Intercept)                  -30.27097 168.67754
purification_bead_cleanupYES -56.55773 153.46633
```

Sequencing Cycles

```
Linear mixed model fit by REML. t-tests use Satterthwaite's method [
lmerModLmerTest]
Formula: adjusted_coverage_per_5_million_reads ~ sequencing_cycles + (1 |
    study)
   Data: df

REML criterion at convergence: 10156.8

Scaled residuals:
    Min      1Q  Median      3Q     Max
-6.7164 -0.4633  0.1510  0.6321  2.1198

Random effects:
 Groups   Name        Variance Std.Dev.
 study    (Intercept) 397.1    19.93
 Residual             790.6    28.12
Number of obs: 1065, groups:  study, 10

Fixed effects:
                   Estimate Std. Error        df t value Pr(>|t|)
(Intercept)       -63.01686   27.03661   7.87967  -2.331 0.048574 *
sequencing_cycles   0.48862    0.07226   7.84536   6.762 0.000157 ***
---
Signif. codes:  0 '***' 0.001 '**' 0.01 '*' 0.05 '.' 0.1 ' ' 1

Correlation of Fixed Effects:
            (Intr)
sqncng_cycl -0.969
```

```
                        2.5 %      97.5 %
.sig01             11.4071590  30.5314926
.sigma             26.9583255  29.3593585
(Intercept)      -115.4170487 -10.7963225
sequencing_cycles   0.3489083   0.6285712
```
